# Co-created Future Scenarios as a Tool to Communicate Sustainable Development in Coastal Communities in Palawan, Philippines

**DOI:** 10.3389/fpsyg.2021.627972

**Published:** 2021-11-22

**Authors:** Isabell Richter, Joel Sumeldan, Arlene Avillanosa, Elizabeth Gabe-Thomas, Lota Creencia, Sabine Pahl

**Affiliations:** ^1^Department of Psychology, University of Plymouth, Plymouth, United Kingdom; ^2^Institute of Psychology, Norwegian University of Science and Technology, Trondheim, Norway; ^3^Department of Fisheries and Aquaculture, Western Philippines University, Puerto Princesa, Philippines; ^4^Plymouth Marine Laboratory, Plymouth, United Kingdom; ^5^Institute for the Psychology of Cognition, Emotion and Methods, University of Vienna, Vienna, Austria

**Keywords:** future scenarios, intentions, consideration of future consequences, coastal communities, co-creation

## Abstract

Scenarios can be used to communicate potential future changes and engage and connect different audiences in exploring sustainable solutions. Communicating scenarios using creative visualisation, co-creation and a focus on local contexts are especially promising. This research is conducted on the island of Palawan in the Philippines as part of the GCRF Blue Communities project. With a quasi-experimental design, we investigate the psychological and emotional effects of the engagement with future scenarios as a tool for communicating sustainability. Together with local stakeholders and community members, three distinct, locally relevant scenario narratives (Business as Usual, Best Case, and Worst Case) have been co-created. Subsequently, a sample of *N* = 109 local high school students was asked to creatively engage with these scenario narratives. Intentions to engage in sustainable behaviour, perceived behavioural control, ascription of responsibility, consideration of future consequences, six basic emotions and connectedness to place were assessed before and after the activity via paper-pencil administrated questionnaires. A mixed-model analysis showed significant increases in intentions to engage in sustainable behaviour, however, this increase disappeared when consideration of future consequences was added as a covariate, suggesting a mediating effect. The level of consideration of future consequences also increased significantly after engaging with any of the future scenarios, which questions the common interpretation of consideration of future consequences as a trait variable. Perceived behavioural control significantly increased following the engagement with each of the scenarios whereas ascription of responsibility and connectedness to place did not show any changes. Overall, the two most emotion-evoking scenarios, Best Case Scenario and Worst Case Scenario, turn out as superior over the Business as Usual Scenario, which points to the relevance of emotional framing for effective messaging in our sample. This is the first systematic, quantitative assessment of the effects of future scenarios as a communication tool.

## Introduction

The importance of achieving the 2030 Agenda of Sustainable Development is globally recognised and 17 goals (SEGs) have been formulated, representing 17 areas of importance. What has been discussed less so far is how these 17 global goals are to be translated and communicated on a local scale; ideally by developing and evaluating tailor-made strategies for different locations and challenges.

Facing social and economic struggles, many communities around the world are balancing the conflict between their everyday needs and the needs of future generations, possibly compromising environmental sustainability. This is because making sustainable choices might result in immediate economic disadvantages, which can have particularly severe consequences in the Global South. Most subsistence communities are forced to prioritise day-to-day adaptation to an ever-changing environment, which they closely depend on for food, health and livelihood over long term strategies for sustainable development (Kroll et al., 2019; Scharlemann et al., 2020). Research on human perceptions and behaviour systematically over-represents university samples from industrialised Western countries, leading to an information deficit around decisions, behaviour and communication strategies of people who live in developing regions and are directly affected by the conflict between everyday subsistence and sustainable development.

Future scenarios are a popular means to communicate the potential prospects of climate change (for example see IPCC, 2021) and might be a means to engage communities and policy makers around the world in sustainable development. The psychological and emotional effects of scenarios as communication tool, especially on lay audiences, are still under researched and direct links between future scenario communication and sustainable action are questionable (Dieckmann et al., 2017; Guilbeault et al., 2018; Xexakis and Trutnevyte, 2021). Very complex graphs or tables can even lead to confusion and reactance (McMahon et al., 2015). It is therefore recommended to customise formats of future scenarios to the audience (Corner et al., 2018; Xexakis and Trutnevyte, 2021), for example with the use of non-technical solutions such as narratives or visuals. These formats, however, still lack thorough evaluation for their effects. In this work, we aim to evaluate the psychological and emotional effects of engaging with future scenario narratives and the co-creation of future scenario visuals. To increase the significance of this technique beyond the Western context, this study has been conducted in Palawan, the Philippines, an area that does not only represent a region of particular ecological vulnerability, but also provides insights into understudied communities (Henrich et al., 2010a,b). This research is part of the GCRF Blue Communities project^1^ which aims to support sustainable co-management of marine resources whilst protecting marine ecosystems and enabling alternative livelihoods via capacity building as a collaborative approach between the United Kingdom and South-East Asian countries.

This is the first systematic assessment of the psychological and emotional effects of differently framed scenarios.

## Future Scenarios: Forms and Application

Developing alternative scenarios to depict different variations of how the future might look like is not new (Fontela and Hingel, 1993) and is used in scientific-, socio- political-, business-, and communication contexts. As Schoemaker (1995) points out in his book, scenarios can be both an outcome of traditional, numeric data simulation or of “soft” data, like cultural frameworks, community structures, political regulations, values and human behaviour, integrating quantitative and qualitative methodologies. The scenarios themselves can take different forms: They can be presented traditionally in the form of graphs or tables, but also as narratives (Steenberg et al., 2019), drawings (Löfström and Klöckner, 2019), infographics, (augmented) photographs (see Tress and Tress, 2003; Sheppard, 2012), GIS-maps (Dockerty et al., 2005) or in virtual reality (Lovett et al., 2002).

Natural scientists develop precise prospects on a variety of dimensions such as levels of carbon emissions, nitrogen in the atmosphere, or fish stocks (Fernandes et al., 2015; Queirós et al., 2016). Another form of future scenario is developed by the International Panel for Climate Change (IPCC). The IPCC’s end-of-century emission scenarios depict what the world would look like under different, almost antithetic regimes (globalisation vs. regionalisation; conservation vs. economy) (IPCC, 2021). These scientific scenarios are used within scientific frameworks but also consulting socio-political decision making (Sala et al., 2000; Merrie et al., 2018). Businesses including large commercial companies have a history to develop future scenarios to gain economic advantages and enhance their resilience (Schoemaker, 1991, 1995). In participatory workshops around the world, scenarios have been co- developed and used as a communication tool to enrich dialogues or inform local policy making (for examples see Berkhout et al., 2002; Kok et al., 2007, 2015; Kok and van Vliet, 2011; Varela-Ortega et al., 2013; Intergovernmental Panel on Climate Change, 2014). According to anecdotal remarks during these workshops, alternative scenarios might have the potential to engage people with the relationship between the current situation and potential futures (Amer et al., 2013), evoke higher levels of problem awareness and encourage community members and policymakers in solution development (Johnson et al., 2012; Sheppard, 2012) and support the identification of obstacles for change processes, such as finances, governance structures (Kok et al., 2011) or a lack of trust (Tress and Tress, 2003). Scenarios might even contribute to larger scale system change (Moss et al., 2010; Darbas et al., 2011). Systematic, empirical evaluation of potential psychological and emotional effects as well as whether any subsequent behavioural changes are attributable to the scenario work are so far lacking (O’Riordan et al., 2008; Measham et al., 2012).

## Psychological Principles and Biases Driving Sustainable Behaviour Change

There are numerous barriers for climate action (for an overview see Gifford, 2011). The ones that can potentially be addressed with the help of co-created future scenarios include non-accessible, specialist information, ignorance and numbness, psychological distance, and temporal discounting.

### Tailored Communication

Lack of environmental action on individual and communal level is commonly interpreted as the result of an information deficit (Lorenzoni et al., 2007); however, increasing the availability of natural science evidence *per se* (e.g., evidence on climate change effects) has not been found to be a strong, direct trigger of sustainable behaviour change (Whitmarsh, 2011). A range of principles have been discussed to improve the accessibility of communication about sustainability which are based on knowledge about fundamental abilities and constraints of the human brain to perceive time and the future (for an overview see Klöckner, 2015). One of the key recommendations is audience-tailored communication which has the potential to spark environmental action (Moser, 2010, 2014; Mycoo, 2015; Harold et al., 2020). Further, messages are processed more successfully when they are made easy to understand for lay people and experts alike (Behavioural Insights Team, 2010; Center for Research on Environmental Decisions, 2014), emphasise a social dimension (Zlatev et al., 2010; Bain et al., 2012) tell a story or refer to a well-known narrative (Garb et al., 2008; Pahl and Bauer, 2013; Nabi and Green, 2015) and consider the human preference of visual information processing (Nicholson-Cole, 2005; Sheppard, 2012; Corner et al., 2015). As an example, Sheppard (2005, 2008) and Sheppard et al. (2011) created realistic imagery depicting the future of local landscapes as a means of engaging community members with climate change to support sustainable regional development. The researchers report that community members responded with increased engagement, understanding and joint environmental decision making, however, they point out that systematic evidence on psychological and emotional effects is needed to understand these processes better.

### Emotional Engagement

Ignorance and numbness are common barriers of climate action. Communication is considered impactful, persuasive and lead to action if it evokes emotions (Pooley and O’Connor, 2000; Slovic et al., 2002). This holds for both positive and negative emotions (O’Keefe and Jensen, 2009; Nabi and Myrick, 2019), discrete emotions and transforming emotions [emotional flow, Nabi and Green (2015) and Nabi et al. (2018)]. Positive emotions such as hope have been found to encourage pro-environmental behaviour (Ojala, 2012), but only is specific actions are included in the message (Hornsey and Fielding, 2016). Negative emotions such as fear or anger as reactions to a story or visual usually evoke strong responses which could be used as catalyst (Pestridge, 2017; Hornsey and Fielding, 2020). In both cases, it is vital to combine the (positive or negative) message with action information to facilitate the feeling of self-efficacy (Tannenbaum et al., 2015) and thereby prevent unwanted responses such as ignorance or rejection. Previous studies provide contradicting evidence regarding emotional framing and behaviour change. Feinberg and Willer (2011) found reactance effects caused by (negative) emotional scenario framing whereas Nabi et al. (2018) claims that positive and negative framing of climate change messages leads to attitude and behaviour change, mediated by emotions like hope and fear. According to the Extended Dual Process Model, individuals only take emotionally motivated action if they feel able to undertake the necessary action that can avoid the threat (Witte, 1992). For scenarios, this implies that positively or negatively framed future visions such as Best- or Worst-Case Scenarios might have stronger effects on motivation and behaviour compared to an emotionally neutral prospect if combined with specific action advice.

### Consideration of Future Consequences

One core psychological mechanism impeding sustainable engagement is that the impacts of many pressing environmental problems, such as climate change and sea-level rise, have been found to be perceived as “psychologically distant.” This means that these threats are seen as geographically distant (Lorenzoni et al., 2006, 2007), happening at a point in time that is too far away to relate to Pahl et al. (2014), and happening to others rather than to ourselves (Spence et al., 2012; Myers et al., 2013). Researchers are exploring ways to overcome this psychological distance to encourage sustainable behaviour change, for example, through proximisation of climate change by presenting people with information about local climate change effects (Spence and Pidgeon, 2010; Brügger et al., 2015), by using tangible time horizons (Tonn et al., 2006) and by communicating via common narratives or experiential visualisation, such as the Future Delta 2 video game (Dulic et al., 2016; Breves and Schramm, 2021) or the ecosystem simulation game ECO (Fjællingsdal and Klöckner, 2019). These recommendations could be combined in co-created future scenarios. As one core characteristic of future scenarios is the temporal dimension, they might have the potential to help people overcome the temporal discounting bias (assuming that environmental problems will only take place in the far future) and to start taking more responsibility for their current actions. In a meta-analysis, Milfont et al. (2012) show that people considering the future outcomes of their actions more, behave more environmentally friendly. Stable interindividual as well as intercultural differences in the level of how much people consider the future consequences can explain variance in actual environmental engagement (Bain et al., 2015). This concept has been conceptualised as a trait measure, called consideration of future consequences (CFC; Strathman et al., 1994; Joireman et al., 2012; Arnocky et al., 2014). It represents the level of how much people think in long-or short time horizons and consequently adapt their actions. So far, no research is available demonstrating if or how CFC could be strengthened to benefit sustainable development.

## Current Study

In the current research, we sought to explore the effects of co-created future scenarios as a communication tool. More precisely, we assess changes in a selection of psychological and emotional variables as a consequence of engaging creatively with a future scenario that was either emotionally framed (Best Case Scenario and Worst Case Scenario) or neutrally framed (Business as Usual Scenario).

The study sample falls into the category of non-WEIRD societies, which have been found to differ from WEIRD samples in several characteristics like risk perception, decision making or moral reasoning (Henrich et al., 2010a,b; Arnett, 2016), requiring suitable measures and methods. The study design as well as the survey questionnaire were co-created by resident and international researchers and adapted to local circumstances, resulting in a unique set of variables. The commitment of the Blue Communities project to deliver capacity-building points toward a special interest in variables that represent agency and means to drive and manage sustainable development on the community level.

### Selected Constructs and Measures

The variables we identified during stakeholder consultations ahead of this study as well as borrowed from popular theories from environmental psychology such as the Theory of Planned Behaviour and the Norm Activation Model (Schwartz, 1977; Ajzen, 1991). Intentions to engage in sustainable behaviour (Int_sust), consideration of future consequences (CFC), perceived behavioural control (PBC), ascription of responsibility (ASC), connectedness to place and emotions (worry, hope, fear, anger, curiosity, and empowerment) have been included.

As a key determinant of pro-environmental behaviour, an adapted measure for behavioural intentions has been included with one item (*In the near future, I want to engage in more work that helps my community to be sustainable*) (Ajzen, 1991). As behaviour change for sustainable development in our study site encompasses a large variety of actions (e.g., selective fishing methods or uptake of alternative livelihoods), we opted for a more general term. It is to note that this stands in contrast with the recommendation by Kaiser and Gutscher (2003) to adapt the specific level of the behavioural intention measure with the behaviour in question. In our case, we decided to opt for the general measure to allow individual interpretation of the item and keep the survey short.

Judgements of how easy behavioural performance is perceived are reflected in *perceived behavioural control* (PBC). We included one item for this construct (*I think it is difficult to do something for my community as an individual*, reversed) adapted from Ajzen (1991). Especially in the context of sustainable practices, behaviours might seem complicated or unfamiliar, leaving people with low confidence to engage in them (Armitage and Conner, 2001; Lorenzoni et al., 2007; Simmons and Fielding, 2019). According to our consultations, local stakeholders and communities often struggle with a sense of disempowerment and helplessness, making PBC a key construct to include.

The feeling of being responsible for negative consequences if not acting pro-socially is represented by the psychological construct *ascription of responsibility* (ASC) (De Groot and Steg, 2009). Traditionally part of the Norm Activation Model (Schwartz, 1977), ASC is known as an indirect predictor of intentions for pro-environmental behaviour (Bamberg and Möser, 2007; Klöckner, 2013; Han, 2014). Feeling responsible for the sustainable development of the community has turned out to be a key theme that can spark sustainable behaviour change (Kaiser and Shimoda, 1999), especially in small communities in low-income countries (Simmons and Fielding, 2019). We included one item (*I don’t feel responsible for the problems of my community*) adapted from Doran and Larsen (2016).

When it comes to sustainable change-making, considering the future consequences of our actions today is of key relevance. The trait measure “*Consideration of Future Consequences*” (CFC) can explain inter-individual differences in future-oriented behaviour and reflects the extent to which people consider the distant and imminent consequences of their behaviour (Arnocky et al., 2014; Murphy and Dockray, 2018). This concept is related to the expression of pro- environmental, -social and -health related intentions and involvement (Joireman et al., 2001, 2012). We included a shortened, five-item CFC scale suggested by Joireman et al. (2012) measuring the Future dimension of CFC (example item: *In the near future, I want to engage in more work that helps my community*).

*Feeling connected* to a particular place or region has been found to drive pro-environmental behaviours such as sustainable land management, clean-ups, recycling or water conservation (Vaske and Kobrin, 2001; Scannell and Gifford, 2010). The nature and size of this place can vary, which means that for some people it might be the own property (Stedman, 2002), for some it is a national park (Halpenny, 2006), for some it is their country (Laczko, 2005; Bonaiuto et al., 2006; Gustafson, 2009) and for some an even wider area such as the planet as a whole, which is reflected in our measurement levels. To assess on what level of abstraction our participants experience place connectedness, we asked them how connected they feel to their city, region, country and the world as a whole (adapted from Williams and Vaske, 2003).

All above-mentioned constructs were measured on a 1 (*Strongly disagree*) – 5 (*Strongly agree*) Likert scale.

People’s decision making is also influenced by their *emotions*, which is known as affect heuristic (Slovic et al., 2002). According to Sheppard (2005), being exposed to scenarios leads to affective responses and the urge to adapt and prepare for the future, however, the author is not specific on the type of emotions. As we expect a range of different emotions to be evoked, also depending on the type of scenario developed, we included six basic emotions, worry, hope, fear, anger, curiosity and empowerment, measured via the Positive and Negative Affect Schedule (PANAS, *Thinking about the future makes me feel*…) (Watson et al., 1988). The selection of emotions was based on literature documenting the effects of climate change scenario communication (Nicholson-Cole, 2005; Sheppard, 2005; Healey and Hodgkinson, 2008).

## Research Questions

RQ1: How does engaging with future scenarios affect Int_sust, CFC, PBC, ASC, Con_place, and emotions?

RQ2: Does engaging with emotionally framed scenarios affect Int_sust, CFC, PBC, ASC, Con_place, and emotions differently as compared to the non-emotionally framed scenario?

RQ3: Does the initial level of CFC influence the effect that future scenarios have on Int_sust, PBC, and ASC?

## Materials and Methods

### Scenario Development

The Blue Communities project engages with communities from three regions across Palawan (Puerto Princesa, Aborlan, and Taytay) of which one (Taytay) was selected as a focus area for this study. In collaboration with 23 local stakeholders from various sectors such as the local government, NGOs, representatives of fisheries, aquaculture and tourism, three scenarios were developed (see Table 1 in Supplementary Document 4 for list of attendees; see Supplementary Document 4 for more details on this process). The first scenario, Business as Usual (BAU), represents the continuation of the current situation and its developments into the future (see Supplementary Document 1). Under this scenario, the current local problems of the local community such as illegal fishing, mangrove cutting and commercial fishing vessel intrusion were narrated and the most likely future outcomes in the next 15 years were depicted. The second scenario, termed here as Worst Case Scenario (WCS), represents the communities’ least desirable future (see Supplementary Document 2). In this scenario, current developments have been driven toward a negative extreme through the narrative: steeply declining fish stocks and dead coral reefs, malnourished children, epidemic diseases and ubiquitous pollution. The third scenario is a depiction of the communities’ most desirable future, the Best Case Scenario (BCS) (see Supplementary Document 3). This scenario narrates how through management interventions, a sustainable future was achieved including for example well performing officials, successful mangrove restoration, coral reef protection and sustainable fishing practices. All three scenarios depicted a version of the future in 15 years’ time, following the recommendations to use a human time horizon (Pahl, 2010; Pahl et al., 2014). The development of the scenarios followed the principle of participatory research by Green et al. (2003), according to which the research process is gradually co-shaped by researchers and participants, and scenarios were elicited with group work manuals adapted from Mansfield (2018) (exact task instructions in the Appendix).

The rich volume of information provided by the stakeholders was collaboratively synthesised by the research team and turned into three coherent narratives (see Supplementary Documents 1–3). As a common element, all three narratives revolved around one protagonist, representing a stereotypical local family.

### Design and Procedure

**FIGURE 1:**
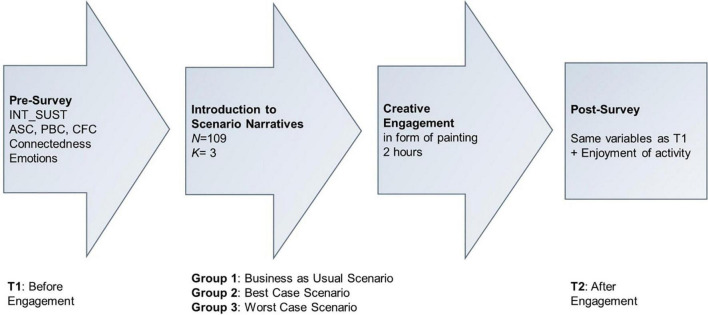
Illustration of steps, sample size, and group structure of the study’s quasi-experimental design.

**FIGURE 2:**
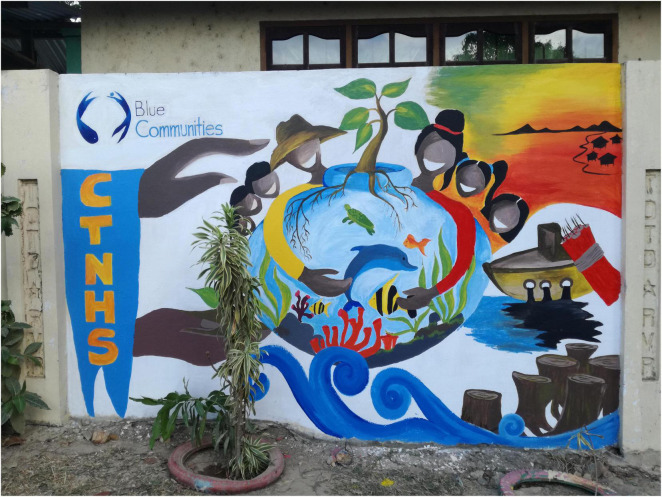
Example of the Business as Usual Scenario drawing, converted into a mural painting in Taytay town after the study was conducted.

**FIGURE 3:**
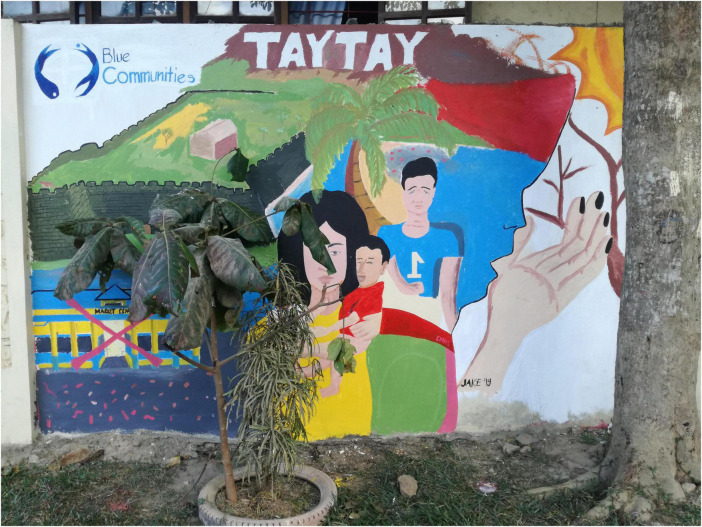
Example of the Worst Case Scenario drawing, converted into a mural painting in Taytay town after the study was conducted.

**FIGURE 4:**
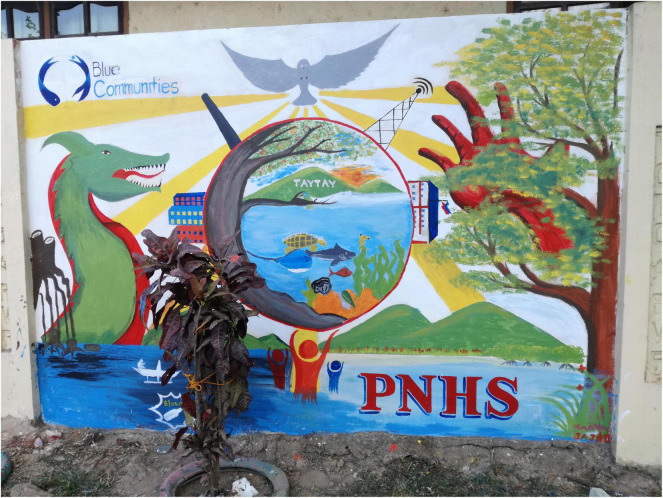
Example of the Best Case Scenario drawing, converted into a mural painting in Taytay town after the study was conducted.

The study design was a 2 (time: pre, post) × 3 (scenarios: BC, BAU, WC) mixed-design (see Figure 1). The study took place in September 2019 over the course of 1 day, starting in the morning with the general registration as well as explanations on the general nature and ethics of the study given by the local head researcher (AA). Subsequently, the first part of the survey (please see Supplementary Document 5, Part 1) was administered in form of a paper and pencil survey, which they had to fill out quietly and seated separately from each other. In case of difficulties with filling out the survey, trained local facilitators supported the participants without influencing their answers. Subsequently, the students were divided into three groups and instructed to move to the designated separate classroom. Each group was accompanied by two researchers, one of which was responsible for reading out and discussing one of the scenario narratives, while the second researcher had a supervisory role. After the three scenario narratives were read out to the respective groups, the students were provided with drawing equipment and instructed to use the following 2 h to discuss and illustrate the narrative they just heard in small groups of 3–5. Examples of the scenario drawings are shown in Figures 2–4. After 2 h spent drawing within their groups the second part of the survey was administered (please refer to Supplementary Document 5, Part 2).

### Sample

The participants (*N*_1_ = 109) were recruited via their teachers from Central Taytay National High School in the municipality of Taytay and the study took place during normal school hours. Slightly more girls (52.3%) than boys (44.7%) participated and all of them came from the Philippines (of which 98% grew up in the study area) originally. Their age ranged between 12 and 18 (*M* = 16.37) and they visited 7th to 12th grade.

### Data Analysis

For the data analysis, initial checks were carried out for all analyses in terms of outlier analysis, checks for normality, homogeneity of variance, multicollinearity and independent observations. Cronbach’s Alpha across the five CFC items has been calculated and can be regarded as questionable with α = 0.64. However, Nunnally (1978) states that Alphas slightly lower than α = 0.70 can be accepted when a small number of items is used or if the research is using under-researched samples or measurements, which is the case here. Therefore, we proceeded to calculate one mean score across the five CFC items for each participant. We also report effect sizes, using (Cohen, 2013) conventions of η^2^ = 0.01 as small, η^2^ = 0.06 as medium and η^2^ = 0.14 as large.

A Mixed Model analysis was conducted for behavioural Int_sust, CFC, PBC, ASC as well as aggregated emotions with the different scenarios as between-group variable and controlling for the level of enjoyment of activity^2^, gender and age. To investigate the initial level of CFC or emotions affects the reaction to the scenario intervention, CFC was added as a covariate into the Mixed Model investigating Int_sust, PBC, and ASC. To receive a more detailed picture on emotional reactions, single mixed model analyses were performed on each emotion (hope, curiosity, empowerment, anger, worry, and fear) separately.

## Results

### Intentions to Engage in Sustainable Behaviour

**FIGURE 5:**
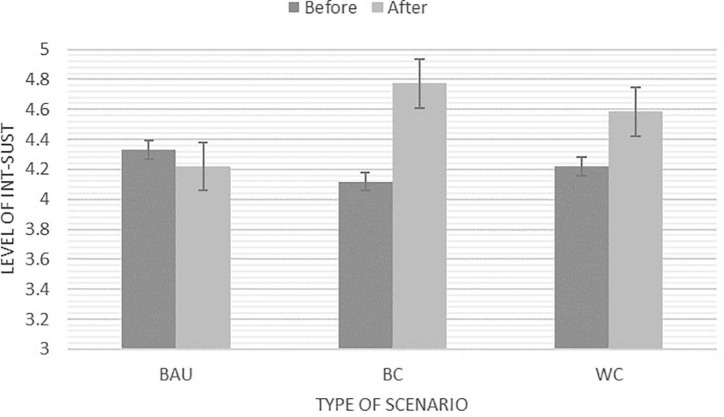
Estimated marginal means for Int_sust before and after the creative engagement with three different types of scenarios: BAU, Business as Usual; BC, Best Case Scenario; and WC, Worst Case Scenario. NB: *Y*-axis adapted for illustration purposes. The Likert scale ranges from 1 (*Strongly disagree*) – 5 (*Strongly agree*).

There was a significant main effect of Int_sust across the two time points, *F*(1,97) = 21.79, *p* < 0.001, η^2^ = 0.18 (*M*_*before*_ = 4.21*; SE_*before*_* = 0.06*;* to *M*_*after*_ = 4.53, *SE*_*after*_ = 0.06). In addition, we also found a significant interaction between time and scenarios *F*(2,97) = 10.98, *p* < 0.001; η^2^ = 0.19. Following up this interaction, there was no significant change in the Business as Usual Scenario group from time 1 to time 2. However, the mean scores for both, the Worst Case Scenario group and the Best Case Scenarios group increased significantly over time. Visual inspection of the estimated marginal means revealed that the biggest changes could be recorded for the Best Case Scenario group, however, the increase was not significantly larger than the increase for the Worst Case Scenario group (see Figure 5).

### Consideration of Future Consequences

**FIGURE 6:**
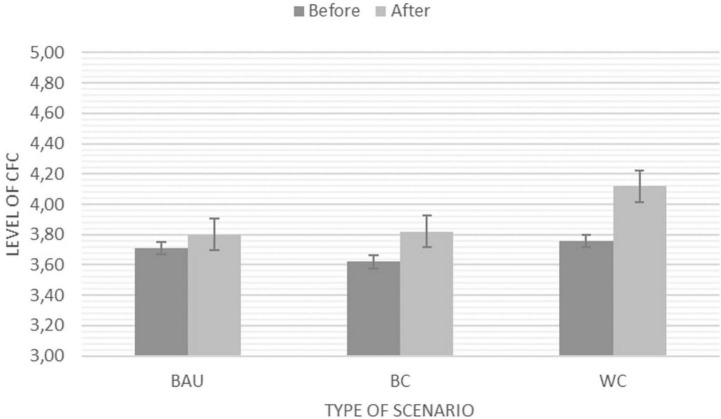
Estimated marginal means for CFC before and after the creative engagement with three different types of scenarios: BAU, Business as Usual; BC, Best Case Scenario; and WC, Worst Case Scenario. NB: *Y*-axis adapted for illustration purposes. The Likert scale ranges from 1 (*Strongly disagree*) – 5 (*Strongly agree*).

We observed a slightly different pattern for CFC as there was a significant main effect across the two time points, *F*(1,93) = 30.04, *p* < 0.001, η^2^ = 0.24, (*M*_*before*_ = 3.72*; SE_*before*_* = 0.45*; M_*after*_* = 3.95, *SE*_*after*_ = 0.47) but no significant interaction between time points and activity (see Figure 6). This indicates that all types of scenarios lead to a greater level of CFC with the Worst Case Scenario recording the strongest change upon visual inspection, however, not significantly stronger than the other two scenarios (see Figure 6).

### Consideration of Future Consequences as Covariate

**FIGURE 7:**
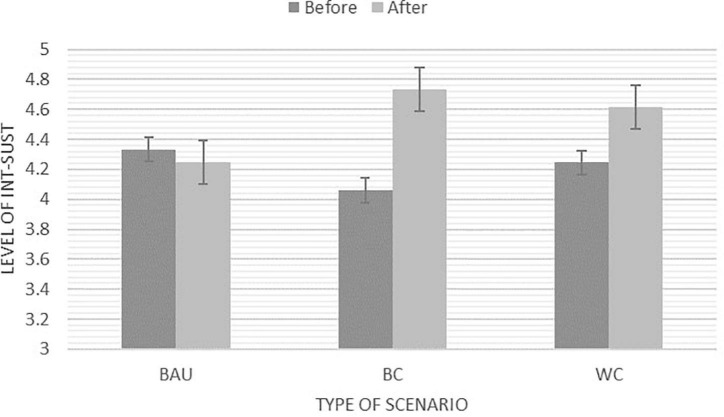
Significant interaction between scenarios after adding CFC as covariate. NB: *Y*-axis adapted for illustration purposes. The Likert scale ranges from 1 (*Strongly disagree*) – 5 (*Strongly agree*).

If we control for the initial level of CFC, the significant main effect of Int_sust disappears, indicating that the change that we observed before and after engaging with the scenarios depends more on the initial level of CFC than the type of scenario. The crossover interaction between time and activity remains significant *F*(2,92) = 9.51, *p* < 0.001; η^2^ = 0.17, indicating that Int_sust is increasing in the Best- and Worst Case Scenario whereas it remains relatively stable in the Business as Usual Scenario as visualised in Figure 7.

### Perceived Behavioural Control

Perceived behavioural control increased over time *F*(1,98) = 3.96, *p* < 0.05, η^2^ = 0.04, (*M*_*before*_ = 3.04*; SE_*before*_* = 1.19*; M_*after*_* = 3.30, *SE*_*after*_ = 1.27), but similar to CFC, no interaction between the type of scenario and the increase of PBC was observed. Due to the non-normality of the perceived behavioural control variable, we suggest applying a more conservative *p*-value of *p* = 0.01 which indicates treating this effect with caution.

### Emotions

#### Aggregated Positive Emotions

**FIGURE 8:**
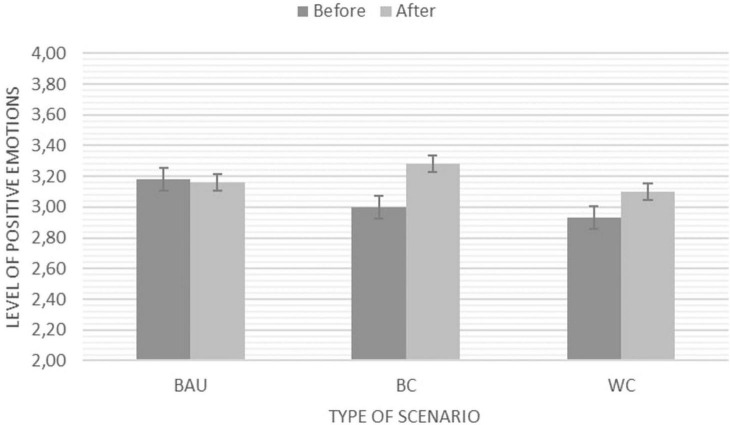
Significant main effect and interaction for positive emotions before and after engaging with one of the three scenarios (BAU, Business as Usual; BC, Best Case Scenario; and WC, Worst Case Scenario). NB: *Y*-axis adapted for illustration purposes. The Likert scale ranges from 1 (*Strongly disagree*) – 5 (*Strongly agree*).

There was a significant main effect of positive emotions across the two time points, *F*(1,96) = 12.43, *p* = 0.001, η^2^ = 0.15 indicating stronger positive emotions (on aggregated level) after the scenario engagement *M*_*before*_ = 3.02*; SE_*before*_* = 0.04*;* to *M*_*after*_ = 3.17, *SE*_*after*_ = 0.04. In addition, we also found a significant interaction between time and scenarios *F*(2,96) = 4.03, *p* = 0.021; η^2^ = 0.08. Following up this interaction indicated that there was no significant change in the Business as Usual Scenario group from time 1 to time 2, whereas the Best Case Scenario and the Worst Case Scenario lead to significant increases in positive emotions with the Best Case Scenario showing the strongest effects as visualised in Figure 8.

#### Aggregated Negative Emotions

Aggregated negative emotions did not show any significant changes over the course of the engagement with the future scenarios *F*(1,97) = 0.05, *p* = 0.953, η^2^ = 0.001; *M*_*before*_ = 2.24*; SE_*before*_* = 0.06*;* to *M*_*after*_ = 2.29, *SE*_*after*_ = 0.06.

#### Individual Emotions

The General Linear Model across each of the six measured emotions individually shows that we can identify significant changes in three emotions: hope, empowerment, and anger.

Hope showed a significant main effect *F*(1,98) = 12.86, *p* = 0.001, η^2^ = 0.12 with higher average values of hope after the engagement of the scenario than before (*M*_*before*_ = 3.95*; SE_*before*_* = 0.08*;* to *M*_*after*_ = 4.27, *SE*_*after*_ = 0.07). Visual inspection points toward the Best Case Scenario inducing the strongest increase, albeit not significantly different from the Worst Case Scenario and Business as Usual.

**FIGURE 9:**
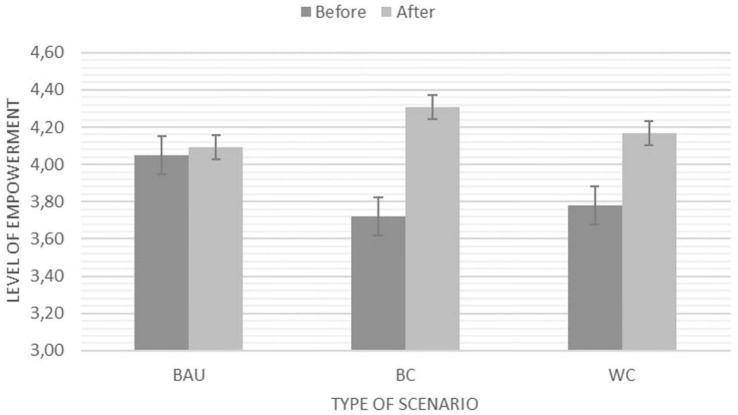
Significant main effect and interaction for the feeling of empowerment before and after engaging with one of the three scenarios (BAU, Business as Usual; BC, Best Case Scenario; and WC, Worst Case Scenario). NB: *Y*-axis adapted for illustration purposes. The Likert scale ranges from 1 (*Strongly disagree*) – 5 (*Strongly agree*).

Both a significant main effect *F*(1,96) = 15.89, *p* < 0.001, η^2^ = 0.14 (*M*_*before*_ = 3.85*; SE_*before*_* = 0.07*;* to *M*_*after*_ = 4.19, *SE*_*after*_ = 0.07) and a significant interaction *F*(1,98) = 3.25, *p* = 0.04, η^2^ = 0.06 were found for the feeling of empowerment after the engagement with our three scenarios. We need to be cautious with interpreting this particular interaction, as the before-level in the Business as Usual group deviates from the before-level of the other two scenarios as can be seen in Figure 9, albeit not significantly.

**FIGURE 10:**
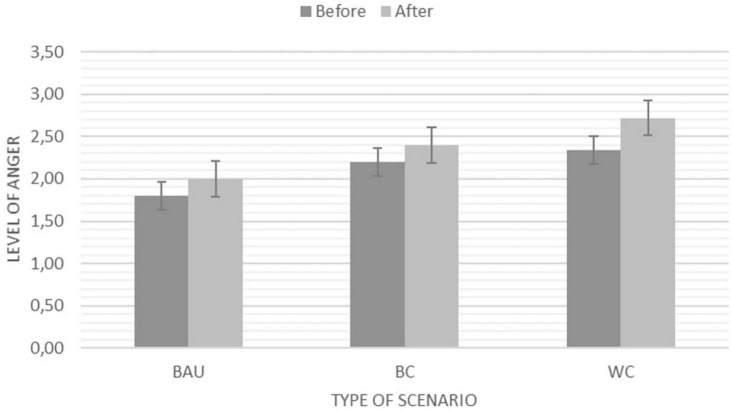
Significant main effect for the feeling of anger before and after engaging with one of the three scenarios (BAU, Business as Usual; BC, Best Case Scenario; and WC, Worst Case Scenario). NB: *Y*-axis adapted for illustration purposes. The Likert scale ranges from 1 (*Strongly disagree*) – 5 (*Strongly agree*).

We found a significant main effect for the feeling of anger after the engagement with the future scenarios. Individual inspection of the values shows that the Worst Case Scenario increased the most, however, not significantly more than the Best case and the Business as Usual Scenario (see Figure 10). Overall, we can note that the levels of anger were relatively low compared to other emotions. Curiosity, worry and fear did not significantly change throughout the activity.

### Additional Analyses

ASC did not significantly change across time *F*(1,98) = 2.73, *p* = 0.102, η^2^ = 0.03; *M*_*before*_ = 3.79*; SE_*before*_* = 0.09*;* to *M*_*after*_ = 3.97, *SE*_*after*_ = 0.10 and no meaningful differences could be observed between before and after our intervention for connectedness to place, i.e., connectedness to the city *F*(1,98) = 2.55, *p* = 0.11, η^2^ = 0.03; *M*_*before*_ = 3.96*; SE_*before*_* = 0.09*;* to *M*_*after*_ = 4.08, *SE*_*after*_ = 0.09, the region *F*(1,98) = 0.1.08, *p* = 0.30, η^2^ = 0.01; *M*_*before*_ = 3.83*; SE_*before*_* = 0.09*;* to *M*_*after*_ = 3.90, *SE*_*after*_ = 0.09, the country *F*(1,98) = 0.58, *p* = 0.45, η^2^ = 0.006; *M*_*before*_ = 4.11, *SE*_*before*_ = 0.09*;* to *M*_*after*_ = 4.06, *SE*_*after*_ = 0.09 or the world *F*(1,98) = 0.10, *p* = 0.75, η^2^ = 0.001; *M*_*before*_ = 3.69*; SE_*before*_* = 0.09*;* to *M*_*after*_ = 3.71, *SE*_*after*_ = 0.10.

## Discussion

We sought to investigate whether engaging with different types of future scenarios affects people’s behavioural intentions to engage in sustainable behaviour, consideration of future consequences, perceived behavioural control, ascription of responsibility, connectedness to place and emotions.

Overall, we can summarise that engaging with scenarios, especially the emotionally framed ones (Best Case and Worst Case Scenario), led to significant changes most variables we measured and that the construct consideration of future consequences deserves some special attention.

Responding to RQ1 and RQ2, we find increased levels of intentions to engage in sustainable behaviour after our intervention. However, it appears that the Best- and Worst Case Scenario were mainly responsible for this effect. A similar pattern was found for perceived behavioural control and positive emotions. In all these cases we found significant changes in the Worst- and the Best Case Scenarios but no or significantly lesser changes for the Business as Usual scenario. The Business as Usual Scenario was by definition created surprise-free and as close as possible to the realistic vision the community members of Taytay hold about their future. The instruction for the Best- and Worst Case Scenario, however, was to think out of the box and create future visions that drive the current positive (for the Best Case Scenario) or negative (for the Worst Case Scenario) developments of Taytay to the extreme. A possible explanation for the effects we found is that emotionally stimulating scenario narratives (Best- and Worst Case Scenario) are more impactful than narratives that focus on the most probable future which is in line with evidence showing that emotions are a key ingredient of impactful narratives (Pooley and O’Connor, 2000; Loewenstein et al., 2001; Lecheler et al., 2013; Nabi et al., 2018). This is supported by the theory of affect heuristics in decision making (Finucane et al., 2000; Slovic et al., 2002).

Looking closer at the effects the scenarios had on our participants’ emotions, we found that especially positive emotions, hope and empowerment, were affected, but also the feeling of anger increased significantly. Aggregated positive emotions and also the individual measures of hope and empowerment increased after engaging with the Best Case Scenario, a narrative that very positively depicted the future of Taytay, expanding on existing sustainable developments in the region including feasible solutions such as pro-environmental programs and investments. Overall, the Best Case Scenario induces the strongest emotional effects compared to the other two scenarios. This underlines how important it is to use positive language, present realistic solutions and thereby spark people’s optimism.

The participant’s positive emotions also increased after engaging with the Worst Case Scenario, but not with the Business as Usual Scenario, which seems counterintuitive at first but mirror the findings by Nabi and Prestin (2016). Their study on emotionally consistent narratives shows that stories framed positively and including solutions (hope/high efficacy) equally boosted intentions to engage in protective actions as did stories that were framed negatively and without solutions (fear/low efficacy) as compared to emotionally inconsistent narratives. Our scenario narratives mimicked the same structure. In our case, the positive emotions evoked by the apocalyptic scenario might be related to wishes for rehabilitation and reconstruction and the possibility of a new start for the community. As the Worst Case Scenario has been depicted overly negative, the positive emotions could also stem from the assumption that reality will most likely be better than the Worst Case Scenario and that there is still time to change course. Further, sustainability is a topic that is now taught in schools in Palawan, potentially leading to a sense of optimism amongst the children.

The rise of anger as a consequence of engaging with all three of the scenarios might be due to negative future prospects on which children in particular do not have a lot of influence as many problems are caused by the generation before them. This is in line with the finding that ascription of responsibility did not change across the activity indicating that the children did not feel more responsible for the state of their local area. A current lack of environmental law enforcement coupled with the limited allocation of resources to sustainable development projects in Palawan may leave children feeling angry. Adding open questions to elaborate more on the reason for and direction of the anger might have provided more insights. Overall, these findings confirm the assumption that emotionally framed scenarios evoke emotions which might work as a catalyst for intentions to engage in sustainable behaviour (Nabi et al., 2018, 2019).

Particular attention should be paid to RQ3, looking at the effects in the consideration of future consequences which is traditionally interpreted as a stable personality trait. One main hurdle for sustainable behaviour change is the lack of an apparent connection between current behaviour and future consequences (Gifford, 2011; Fauré, 2016; Wittmann and Sircova, 2018). We found that engaging with any of the three future scenarios led to a significant increase in consideration of future consequences. Compared to the other effects in which the emotional scenarios were superior, consideration of future consequences significantly increased across all three scenarios. This might indicate that it is the engagement with scenarios of any kind that helps people to establish a closer connection to the future and therefore consider the consequences of their behaviours more. The significant increase of consideration of future consequences across all conditions also indicates that the interpretation of consideration of future consequences as a stable trait might have to be reinterpreted as it can be manipulated by immersing people with future scenarios. Toepoel (2010) argues that consideration of future consequences is subject to slow changes over the course of life, driven by education or significant life events. However, we identified significant changes after only a few hours. We have reason to assume that creative engagement with future scenarios can have similar effects as significant life events. In contrast to significant life events, engagement with future scenarios can be induced in a single experimental setting. This is relevant evidence on the possibility to increase people’s level of consideration of future consequences by co-creating future scenarios. This effect has not been discovered before and might, together with the increased level of perceived behavioural control after engaging with all types of scenarios provide some leverage for the development of effective communication strategies and eventually for sustainable behaviour change.

The finding that engaging with the future did not change our participants’ connectedness scores shows that this factor remains stable over time and does not interfere with our results or is affected by our intervention. We also found the effects of our scenarios were independent of the demographic characteristics such as gender, age or education of our participants as well as of how much they enjoyed the activity. Especially as our study design was quasi-experimental, it is important to reaffirm in prospective studies that engagement with the scenarios is the reason for the effects we found. Within the GCRF Blue communities project, this experimental setting has been replicated in locally adapted designs in several other study sites. The data collected is currently being analysed and will complement the present study.

## Limitations

From potentially confounding extraneous factors like noises, temperature and weather changes which can affect the participant performance, to variance in interpersonal interactions in the sub-groups the children were working in, there were some factors we could not fully control. This is due to the nature of the study being a field experiment taking place in the ordinary environmental of our participants, on a remote island with limited facilities to conduct controlled research experiments. To limit the impact of interpersonal interactions and conflicts that might have arisen, we controlled for the level of enjoyment of the activity in an additional survey question, which was added as a covariate to the analysis. This variable did not significantly affect any of our analyses, indicating that the activity was perceived as equally enjoyable by all our participants across conditions.

Another limitation is that we did not test several different scenarios of each type (BAU/WC/BC) against each other. Therefore, we cannot clearly say if it was the type of the scenario or the specific narrative leading to our effects. The quasi-experimental design further did not include a control group in which participants did not engage with any future scenario. The question if our effects are merely caused by a creative, social activity can be ruled out by the inter-scenario differences.

Another key limitation is the sample consisting of junior and senior students of the local High School located at the study site, in the city of Taytay. The age cluster between 12 and 18 coincides with puberty and is therefore an emotionally intense and challenging time for most teenagers. Despite the argument that emotion regulation develops across the whole lifespan (Cole, 2014), we are aware of this age cluster being particularly susceptible for emotional triggers (Burnett et al., 2011). Due to this particular age profile of our sample, we advise against generalising this conclusion across all age groups before additional studies have been conducted. Within our sample, we did not find that age differences affected the result patterns. This indicates that at least within the age range covered by our participants, no differences of scenario effects can be observed depending on how old the participants are.

The last limitation concerns the validity of our measurements and responses. To simplify and shorten the survey as much as possible, we only included one item per psychological construct. Ideally, we would have used multi-scale instruments to average out potential measurement errors (Nunnally, 1978). Weighing up the reliability that can be gained by including more items against a possible response error, especially in a children sample, we decided to stick with one item per measurement. The responses could have been influenced by the wish of the children to reply in a socially desirable manner which we attempted to control as much as possible. To encourage the children to provide authentic answers, the facilitators did not interact with the children while they responded to the survey questions because direct interaction could increase social desirability effects (Miller et al., 2015). Furthermore, all participants were notified that the survey will remain anonymous and individual number codes were allocated to each child instead of their names.

## Conclusion

Our study shows that scenarios, that were carefully designed according to the criteria of environmental communication, are powerful tools to communicate about sustainable development. Engaging with co-created, and locally relevant future scenarios significantly increased people’s intentions to engage in sustainable behaviour, their consideration of future consequences, their perceived behavioural control and their positive and negative emotions. Especially emotionally framed scenarios seemed to have a strong effect on people’s motivation to engage in sustainable behaviour change. This underlines the importance to communicate to people not only with factual information but on an emotional level when we want to see change.

Engaging with scenarios also seems to bridge the psychological distance between now and the future and change the individual levels of consideration of future consequences, which is a novel finding that is worth exploring further.

## Data Availability Statement

The raw data supporting the conclusions of this article can be made available on request.

## Ethics Statement

The studies involving human participants were reviewed and approved by the Faculty Research Ethics and Integrity Committee University of Plymouth. Written informed consent to participate in this study was provided by the participants’ legal guardian/next of kin.

## Author Contributions

IR, JS, AA, and SP developed the study design. IR, SP, and EG-T developed the survey questionnaires. JS and AA were responsible for recruitment and correspondence with stakeholders and schools as well as planning of data collection, location, and infrastructure. IR, JS, AA, and LC collected the data and held the stakeholder meetings. IR coded and analysed the data, and wrote the manuscript. JS, AA, LC, and SP gave feedback to the manuscript. SP and LC supervised the project. All authors contributed to the article and approved the submitted version.

## Conflict of Interest

The authors declare that the research was conducted in the absence of any commercial or financial relationships that could be construed as a potential conflict of interest.

## Publisher’s Note

All claims expressed in this article are solely those of the authors and do not necessarily represent those of their affiliated organizations, or those of the publisher, the editors and the reviewers. Any product that may be evaluated in this article, or claim that may be made by its manufacturer, is not guaranteed or endorsed by the publisher.

## Footnotes

1
www.bluecommunities.org

2 The pattern of results and significance levels remain the same if the variables enjoyment of activity, gender and age are added or removed. This rules out that our effects are based on the participants’ enjoying of the group work rather than on the engagement with the scenario content.

## References

[B1] Ajzen I. (1991). The theory of planned behavior. *Organ. Behav. Hum. Decis. Process.* 50 179–211.

[B2] Amer M. Daim T. U. Jetter A. (2013). A review of scenario planning. *Futures* 46 23–40.

[B3] Armitage C. J. Conner M. (2001). Efficacy of the theory of planned behaviour: a meta-analytic review. *Br. J. Soc. Psychol.* 40 471–499. 10.1348/014466601164939 11795063

[B4] Arnett J. J. (2016). The neglected 95%: why American psychology needs to become less American. *Am. Psychol.* 63 602–614. 10.1037/0003-066X.63.7.602 18855491

[B5] Arnocky S. Milfont T. L. Nicol J. R. (2014). Time perspective and sustainable behavior: evidence for the distinction between consideration of immediate and future consequences. *Environ. Behav.* 46 556–582. 10.1177/0013916512474987

[B6] Bain P. G. Hornsey M. J. Bongiorno R. Jeffries C. (2012). Promoting pro-environmental action in climate change deniers. *Nat. Clim. Change* 2 600–603. 10.1038/nclimate1532

[B7] Bain P. G. Milfont T. L. Kashima Y. Bilewicz M. Doron G. Garðarsdóttir R. B. (2015). Co-benefits of addressing climate change can motivate action around the world. *Nat. Clim. Change* 6 154–157. 10.1038/nclimate2814

[B8] Bamberg S. Möser G. (2007). Twenty years after Hines, Hungerford, and Tomera: a new meta-analysis of psycho-social determinants of pro-environmental behaviour. *J. Environ. Psychol.* 27 14–25. 10.1016/j.jenvp.2006.12.002

[B9] Behavioural Insights Team (2010). *Applying Behavioural Insight to Health.* London: Cabinet Office.

[B10] Berkhout F. Hertin J. Jordan A. (2002). Socio-economic futures in climate change impact assessment: using scenarios as ‘learning machines’. *Glob. Environ. Chang.* 12 83–95. 10.1016/s0959-3780(02)00006-7

[B11] Bonaiuto M. Fornara F. Bonnes M. (2006). Perceived residential environment quality in middle-and low-extension Italian cities. *Eur. Rev. Appl. Psychol.* 56 23–34. 10.1016/j.erap.2005.02.011

[B12] Breves P. Schramm H. (2021). Bridging psychological distance: the impact of immersive media on distant and proximal environmental issues. *Comput. Hum. Behav.* 115:106606. 10.1016/j.chb.2020.106606

[B13] Brügger A. Dessai S. Devine-Wright P. Morton T. A. Pidgeon N. F. (2015). Psychological responses to the proximity of climate change. *Nat. Clim. Chang.* 5:1031. 10.1038/nclimate2760

[B14] Burnett S. Thompson S. Bird G. Blakemore S.-J. (2011). Pubertal development of the understanding of social emotions: implications for education. *Learn. Individ. Differ.* 21 681–689. 10.1016/j.lindif.2010.05.007 22211052 PMC3219830

[B15] Center for Research on Environmental Decisions (2014). *Connecting on Climate: a Guide to Effective Climate Change Communication.* Available Online at: http://ecoamerica.org/wp-content/uploads/2014/12/ecoAmerica-CRED-2014-Connecting-on-Climate.pdf (accessed November 9, 2020).

[B16] Cohen J. (2013). *Statistical Power Analysis for the Behavioral Sciences.* Milton Park: Routledge.

[B17] Cole P. M. (2014). Moving ahead in the study of the development of emotion regulation. *Int. J. Behav. Dev.* 38 203–207. 10.1177/0165025414522170

[B18] Corner A. Shaw C. Clarke J. (2018). *Principles for Effective Communication and Public Engagement on Climate Change A Handbook for IPCC.* Oxford: Climate Outreach.

[B19] Corner A. Webster R. Teriete C. (2015). *Climate Visuals: Seven Principles for Visual Climate Change Communication (Based on International Social Research).* Oxford: Climate Outreach.

[B20] Darbas T. Williams R. Graham S. (2011). Green-changing: a research-based collaboration with a tree-changed rural community. *Rural Soc.* 20 256–265. 10.5172/rsj.20.3.256

[B21] De Groot J. I. Steg L. (2009). Morality and prosocial behavior: the role of awareness, responsibility, and norms in the norm activation model. *J. Soc. Psychol.* 149 425–449. 10.3200/socp.149.4.425-449 19702104

[B22] Dieckmann N. F. Gregory R. Peters E. Hartman R. (2017). Seeing what you want to see: how imprecise uncertainty ranges enhance motivated reasoning. *Risk Anal.* 37 471–486. 10.1111/risa.12639 27667776

[B23] Dockerty T. Lovett A. Sünnenberg G. Appleton K. Parry M. (2005). Visualising the potential impacts of climate change on rural landscapes. *Comput. Environ. Urban Syst.* 29 297–320. 10.1016/j.compenvurbsys.2004.05.004

[B24] Doran R. Larsen S. (2016). The relative importance of social and personal norms in explaining intentions to choose eco-friendly travel options. *Int. J. Tour. Res.* 18 159–166.

[B25] Dulic A. Angel J. Sheppard S. R. (2016). Designing futures: Inquiry in climate change communication. *Futures* 81 54–67. 10.1016/j.futures.2016.01.004

[B26] Fauré E. (2016). *Sustainability Goals Combining Social and Environmental Aspects.* Sweden: KTH Royal Institute of Technology.

[B27] Feinberg M. Willer R. (2011). Apocalypse soon? Dire messages reduce belief in global warming by contradicting just-world beliefs. *Psychol. Sci.* 22 34–38. 10.1177/0956797610391911 21148457

[B28] Fernandes J. A. Kay S. Hossain M. A. R. Ahmed M. Cheung W. W. L. Lazar A. N. (2015). Projecting marine fish production and catch potential in Bangladesh in the 21st century under long-term environmental change and management scenarios. *ICES J. Mar. Sci.* 73 1357–1369. 10.1093/icesjms/fsv217

[B29] Finucane M. L. Alhakami A. Slovic P. Johnson S. M. (2000). The affect heuristic in judgments of risks and benefits. *J. Behav. Decis. Mak.* 13 1–17. 10.1002/(sici)1099-0771(200001/03)13:1<1::aid-bdm333>3.0.co;2-s

[B30] Fjællingsdal K. S. Klöckner C. A. (2019). Gaming green: the educational potential of eco – a digital simulated ecosystem. *Front. Psychol.* 10:2846. 10.3389/fpsyg.2019.02846 31920875 PMC6930897

[B31] Fontela E. Hingel A. (1993). Scenarios on economic and social cohesion in Europe. *Futures* 25 139–154. 10.1016/0016-3287(93)90160-u

[B32] Garb Y. Pulver S. VanDeveer S. D. (2008). Scenarios in society, society in scenarios: toward a social scientific analysis of storyline-driven environmental modeling. *Environ. Res. Lett.* 3:045015. 10.1088/1748-9326/3/4/045015

[B33] Gifford R. (2011). The dragons of inaction: psychological barriers that limit climate change mitigation and adaptation. *Am. Psychol.* 66:290. 10.1037/a0023566 21553954

[B34] Green L. W. George M. A. Daniel M. Frankish C. J. Herbert C. P. Bowie W. R. (2003). “Guidelines for participatory research in health promotion,” in *Community-Based Participatory Research for Health*, eds Minkler M. Wallerstein N. (San Francisco, CA: Jossey-Bass), 52.

[B35] Guilbeault D. Becker J. Centola D. (2018). Social learning and partisan bias in the interpretation of climate trends. *Proc. Natl. Acad. Sci. U.S.A.* 115 9714–9719. 10.1073/pnas.1722664115 30181271 PMC6166837

[B36] Gustafson P. (2009). More cosmopolitan, no less local: the orientations of international travellers. *Eur. Soc.* 11 25–47. 10.1080/14616690802209689

[B37] Halpenny E. A. (2006). Environmental Behaviour, Place Attachment and Park Visitation: A Case Study of Visitors to Point Pelee National Park (Thesis). Available online at: http://hdl.handle.net/10012/718

[B38] Han H. (2014). The norm activation model and theory-broadening: individuals’ decision-making on environmentally-responsible convention attendance. *J. Environ. Psychol.* 40 462–471. 10.1016/j.jenvp.2014.10.006

[B39] Harold J. Lorenzoni I. Shipley T. F. Coventry K. R. (2020). Communication of IPCC visuals: IPCC authors’ views and assessments of visual complexity. *Clim. Chang.* 158 255–270. 10.1007/s10584-019-02537-z

[B40] Healey M. P. Hodgkinson G. P. (2008). “Troubling futures: scenarios and scenario planning for organizational decision making,” in *The Oxford Handbook of Organizational Decision Making*, eds Hodgkinson G. P. Starbuck W. H. (Oxford: Oxford University Press).

[B41] Henrich J. Heine S. J. Norenzayan A. (2010a). Most people are not WEIRD. *Nature* 466:29. 10.1038/466029a 20595995

[B42] Henrich J. Heine S. J. Norenzayan A. (2010b). The weirdest people in the world? *Behav. Brain Sci.* 33 61–83. 20550733 10.1017/S0140525X0999152X

[B43] Hornsey M. J. Fielding K. S. (2016). A cautionary note about messages of hope: focusing on progress in reducing carbon emissions weakens mitigation motivation. *Glob. Environ. Chang.* 39 26–34. 10.1016/j.gloenvcha.2016.04.003

[B44] Hornsey M. J. Fielding K. S. (2020). Understanding (and reducing) inaction on climate change. *Soc. Issues Policy Rev.* 14 3–35.

[B45] Intergovernmental Panel on Climate Change (2014). “Climate change 2014: synthesis report,” in *Proceedings of the Contribution of Working Groups I, II and III to the Fifth Assessment Report of the Intergovernmental Panel on Climate Change*, (Geneva).

[B46] IPCC (2021). “Climate change 2021: the physical science basis,” in *Proceedings of the Contribution of Working Group I to the Sixth Assessment Report of the Intergovernmental Panel on Climate Change*, (Geneva: IPCC).

[B47] Johnson K. A. Dana G. Jordan N. R. Draeger K. J. Kapuscinski A. Olabisi L. K. S. (2012). Using participatory scenarios to stimulate social learning for collaborative sustainable development. *Ecol. Soc.* 17:9.

[B48] Joireman J. Lasane T. P. Bennett J. Richards D. Solaimani S. (2001). Integrating social value orientation and the consideration of future consequences within the extended norm activation model of proenvironmental behaviour. *Br. J. Soc. Psychol.* 40 133–155. 10.1348/014466601164731 11329831

[B49] Joireman J. Shaffer M. J. Balliet D. Strathman A. (2012). Promotion orientation explains why future-oriented people exercise and eat healthy: evidence from the two-factor consideration of future consequences-14 scale. *Pers. Soc. Psychol. Bull.* 38 1272–1287. 10.1177/0146167212449362 22833533

[B50] Kaiser F. G. Gutscher H. (2003). The proposition of a general version of the theory of planned behavior: predicting ecological behavior. *J. Appl. Soc. Psychol.* 33 586–603.

[B51] Kaiser F. G. Shimoda T. A. (1999). Responsibility as a predictor of ecological behaviour. *J. Environ. Psychol.* 19 243–253. 10.1006/jevp.1998.9123

[B52] Klöckner C. A. (2013). A comprehensive model of the psychology of environmental behaviour—a meta-analysis. *Glob. Environ. Chang.* 23 1028–1038. 10.1016/j.gloenvcha.2013.05.014

[B53] Klöckner C. A. (2015). *The Psychology of Pro-Environmental Communication: Beyond Standard Information Strategies.* New York: Palgrave Macmillan.

[B54] Kok K. van Vliet M. (2011). Using a participatory scenario development toolbox: added values and impact on quality of scenarios. *J. Water Clim. Chang.* 2 87–105. 10.2166/wcc.2011.032

[B55] Kok K. Bärlund I. Flörke M. Holman I. Gramberger M. Sendzimir J. (2015). European participatory scenario development: strengthening the link between stories and models. *Clim. Chang.* 128 187–200. 10.1007/s10584-014-1143-y

[B56] Kok K. Biggs R. Zurek M. (2007). Methods for developing multiscale participatory scenarios: insights from southern Africa and Europe. *Ecol. Soc.* 12:8.

[B57] Kok K. van Vliet M. Bärlund I. Dubel A. Sendzimir J. (2011). Combining participative backcasting and exploratory scenario development: experiences from the SCENES project. *Technol. Forecast. Soc. Chang.* 78 835–851. 10.1016/j.techfore.2011.01.004

[B58] Kroll C. Warchold A. Pradhan P. (2019). Sustainable Development Goals (SDGs): are we successful in turning trade-offs into synergies? *Palgrave Commun.* 5:140.

[B59] Laczko L. S. (2005). National and local attachments in a changing world system: evidence from an international survey. *Int. Rev. Soc.* 15 517–528.

[B60] Lecheler S. Schuck A. R. de Vreese C. H. (2013). Dealing with feelings: positive and negative discrete emotions as mediators of news framing effects. *Communications* 38 189–209.

[B61] Loewenstein G. F. Weber E. U. Hsee C. K. Welch N. (2001). Risk as feelings. *Psychol. Bull.* 127 267–286. 11316014 10.1037/0033-2909.127.2.267

[B62] Löfström E. Klöckner C. A. (2019). “Nature in your face: framing co-creative visioning,” in *Paper presented at the ECEEE Summer Study Proceedings*, (Stockholm).

[B63] Lorenzoni I. Leiserowitz A. de Franca Doria M. Poortinga W. Pidgeon N. F. (2006). Cross-National comparisons of image associations with “global warming” and “climate change” among laypeople in the United States of America and Great Britain. *J. Risk Res.* 9 265–281.

[B64] Lorenzoni I. Nicholson-Cole S. Whitmarsh L. (2007). Barriers perceived to engaging with climate change among the UK public and their policy implications. *Glob. Environ. Chang.* 17 445–459. 10.1016/j.gloenvcha.2007.01.004

[B65] Lovett A. Kennaway R. Sünnenberg G. Cobb D. Dolman P. O’Riordan T. (2002). *Visualizing Sustainable Agricultural Landscapes (Vol. 1)*. Abingdon, UK: CRC Press.

[B66] Mansfield D. (2018). *15 Creative Exercises That are Better than Brainstorming. Marketing.* Available Onliune at: https://blog.hubspot.com/marketing/creative-exercises-better-than-brainstorming. (accessed November 9, 2020).

[B67] McMahon R. Stauffacher M. Knutti R. (2015). The unseen uncertainties in climate change: reviewing comprehension of an IPCC scenario graph. *Clim. Chang.* 133 141–154.

[B68] Measham T. Darbas T. Williams R. Taylor B. (2012). Rethinking rural futures: qualitative scenarios for reflexive regional development. *Rural Soc.* 21 176–189. 10.5172/rsj.2012.21.3.176

[B69] Merrie A. Keys P. Metian M. Österblom H. (2018). Radical ocean futures-scenario development using science fiction prototyping. *Futures* 95 22–32.

[B70] Milfont T. L. Wilson J. Diniz P. (2012). Time perspective and environmental engagement: a meta-analysis. *Int. J. Psychol.* 47 325–334. 22452746 10.1080/00207594.2011.647029

[B71] Miller P. H. Baxter S. D. Royer J. A. Hitchcock D. B. Smith A. F. Collins K. L. (2015). Children’s social desirability: effects of test assessment mode. *Pers. Individ. Dif.* 83 85–90. 25870465 10.1016/j.paid.2015.03.039 PMC4392397

[B72] Moser S. C. (2010). Communicating climate change: history, challenges, process and future directions. *Wiley Interdiscip. Rev. Clim. Chang.* 1 31–53. 10.1002/wcc.11

[B73] Moser S. C. (2014). Communicating adaptation to climate change: the art and science of public engagement when climate change comes home. *Wiley Interdiscip. Rev. Clim. Chang.* 5 337–358. 10.1002/wcc.276

[B74] Moss R. H. Edmonds J. A. Hibbard K. A. Manning M. R. Rose S. K. Van Vuuren D. P. (2010). The next generation of scenarios for climate change research and assessment. *Nature* 463 747–756. 20148028 10.1038/nature08823

[B75] Murphy L. Dockray S. (2018). The consideration of future consequences and health behaviour: a meta-analysis. *Health Psychol. Rev.* 12 357–381. 10.1080/17437199.2018.1489298 29902949

[B76] Mycoo M. (2015). Communicating climate change in rural coastal communities. *Int. J. Clim. Chang. Strateg. Manag.* 7 58–75. 10.1108/ijccsm-04-2013-0042

[B77] Myers T. A. Maibach E. W. Roser-Renouf C. Akerlof K. Leiserowitz A. A. (2013). The relationship between personal experience and belief in the reality of global warming. *Nat. Clim. Chang.* 3 343–347. 10.3389/fpsyg.2021.669911 34276492 PMC8284052

[B78] Nabi R. L. Green M. C. (2015). The role of a Narrative’s emotional flow in promoting persuasive outcomes. *Media Psychol.* 18 137–162.

[B79] Nabi R. L. Myrick J. G. (2019). Uplifting fear appeals: considering the role of hope in fear-based persuasive messages. *Health Commun.* 34 463–474. 10.1080/10410236.2017.1422847 29313717

[B80] Nabi R. L. Prestin A. (2016). Unrealistic hope and unnecessary fear: exploring how sensationalistic news stories influence health behavior motivation. *Health Commun.* 31 1115–1126. 10.1080/10410236.2015.1045237 26886401

[B81] Nabi R. L. Gustafson A. Jensen R. (2018). Framing climate change: exploring the role of emotion in generating advocacy behavior. *Sci. Commun.* 40 442–468.

[B82] Nabi R. L. Walter N. Oshidary N. Endacott C. G. Love-Nichols J. Lew Z. J. (2019). Can emotions capture the elusive gain-loss framing effect? a meta-analysis. *Commun. Res.* 47 1107–1130.

[B83] Nicholson-Cole S. A. (2005). Representing climate change futures: a critique on the use of images for visual communication. *Comput. Environ. Urban Syst.* 29 255–273.

[B84] Nunnally J. C. (1978). *Psychometrc Theory*, 2nd Edn. New York: McGrw-Hill.

[B85] Ojala M. (2012). Hope and climate change: the importance of hope for environmental engagement among young people. *Environ. Educ. Res.* 18 625–642. 10.1186/s12913-016-1423-5 27409075 PMC4943498

[B86] O’Keefe D. J. Jensen J. D. (2009). The relative persuasiveness of gain-framed and loss-framed messages for encouraging disease detection behaviors: a meta-analytic review. *J. Commun.* 59 296–316. 10.1080/10810730701615198 17934940

[B87] O’Riordan T. Nicholson-Cole S. A. Milligan J. (2008). Designing sustainable coastal futures. *Twenty First Century Soc.* 3 145–157. 10.1080/17450140802062128

[B88] Pahl S. (2010). “Psychological distance—exploring construal level theory in the context of sustainability,” in *Paper Presented at the BPS Seminar Series Psychology of Sustainability*, (Cardiff)

[B89] Pahl S. Bauer J. (2013). Overcoming the distance: perspective taking with future humans improves environmental engagement. *Environ. Behav.* 45 155–169. 10.1177/0013916511417618

[B90] Pahl S. Sheppard S. R. Boomsma C. Groves C. (2014). Perceptions of time in relation to climate change. *Wiley Interdiscip. Rev. Clim. Chang.* 5 375–388.

[B91] Pestridge E. (2017). *The Role of Shock Imagery in Non-Governmental Organisations and Media Campaigns Surrounding the Rhino Poaching Crisis. (MSc).* Kent: University of Kent.

[B92] Pooley J. A. O’Connor M. (2000). Environmental education and attitudes: emotions and beliefs are what is needed. *Environ. Behav.* 32 711–723. 10.1177/0013916500325007

[B93] Queirós A. M. Huebert K. B. Keyl F. Fernandes J. A. Stolte W. Maar M. (2016). Solutions for ecosystem-level protection of ocean systems under climate change. *Glob. Chang. Biol.* 22 3927–3936. 10.1111/gcb.13423 27396719

[B94] Sala O. E. Chapin F. S. Armesto J. J. Berlow E. Bloomfield J. Dirzo R. (2000). Global biodiversity scenarios for the year 2100. *Science* 287 1770–1774. 10.1126/science.287.5459.1770 10710299

[B95] Scannell L. Gifford R. (2010). The relations between natural and civic place attachment and pro-environmental behavior. *J. Environ. Psychol.* 30 289–297.

[B96] Scharlemann J. P. W. Brock R. C. Balfour N. Brown C. Burgess N. D. Guth M. K. (2020). Towards understanding interactions between sustainable development goals: the role of environment–human linkages. *Sustain. Sci.* 15 1573–1584.

[B97] Schoemaker P. J. H. (1991). When and how to use scenario planning - a heuristic approach with illustration. *J. Forecast.* 10 549–564. 10.1002/for.3980100602

[B98] Schoemaker P. J. H. (1995). Scenario planning - a tool for strategic thinking. *Sloan Manag. Rev.* 36 25–40.

[B99] Schwartz S. (1977). Normative influences on altruism. *Adv. Exp. Soc. Psychol.* 10 221–279. 10.1016/s0065-2601(08)60358-5

[B100] Sheppard S. R. (2005). Landscape visualisation and climate change: the potential for influencing perceptions and behaviour. *Environ. Sci. Policy* 8 637–654. 10.1186/s12868-016-0283-6 27534393 PMC5001212

[B101] Sheppard S. R. (2008). Local climate change visioning: a new process for community planning and outreach using visualization tools. *Plan Canada* 48:36.

[B102] Sheppard S. R. (2012). *Visualizing Climate Change: a Guide to Visual Communication of Climate Change and Developing Local Solutions.* Milton Park: Routledge.

[B103] Sheppard S. R. Shaw A. Flanders D. Burch S. Wiek A. Carmichael J. (2011). Future visioning of local climate change: a framework for community engagement and planning with scenarios and visualisation. *Futures* 43 400–412. 10.1016/j.futures.2011.01.009

[B104] Simmons E. C. Fielding K. S. (2019). Psychological predictors of fishing and waste management intentions in Indonesian coastal communities. *J. Environ. Psychol.* 65:101324. 10.1016/j.jenvp.2019.101324

[B105] Slovic P. Finucane M. Peters E. MacGregor D. G. (2002). Rational actors or rational fools: implications of the affect heuristic for behavioral economics. *J. Socio Econ.* 31 329–342.

[B106] Spence A. Pidgeon N. (2010). Framing and communicating climate change: the effects of distance and outcome frame manipulations. *Glob. Environ. Chang.* 20 656–667.

[B107] Spence A. Poortinga W. Pidgeon N. (2012). The psychological distance of climate change. *Risk Anal. Int. J.* 32 957–972. 10.1111/j.1539-6924.2011.01695.x 21992607

[B108] Stedman R. C. (2002). Toward a social psychology of place: predicting behavior from place-based cognitions, attitude, and identity. *Environ. Behav.* 34 561–581.

[B109] Steenberg J. W. Duinker P. N. Creed I. F. Serran J. N. Ouellet Dallaire C. (2019). Alternative scenarios for the future of the Canadian boreal zone1. *Environ. Rev.* 27 185–199. 10.1139/er-2018-0062 33356898

[B110] Strathman A. Gleicher F. Boninger D. S. Edwards C. S. (1994). The consideration of future consequences: weighing immediate and distant outcomes of behavior. *J. Pers. Soc. Psychol.* 66 742–752. 10.1037/0022-3514.66.4.742

[B111] Tannenbaum M. B. Hepler J. Zimmerman R. S. Saul L. Jacobs S. Wilson K. (2015). Appealing to fear: a meta-analysis of fear appeal effectiveness and theories. *Psychol. Bull.* 141 1178–1204. 10.1037/a0039729 26501228 PMC5789790

[B112] Toepoel V. (2010). Is consideration of future consequences a changeable construct? *Pers. Individ. Dif.* 48 951–956. 10.1016/j.paid.2010.02.029

[B113] Tonn B. Hemrick A. Conrad F. (2006). Cognitive representations of the future: survey results. *Futures* 38 810–829.

[B114] Tress B. Tress G. (2003). Scenario visualisation for participatory landscape planning—a study from Denmark. *Landsc. Urban Plan.* 64 161–178. 10.1016/s0169-2046(02)00219-0

[B115] Varela-Ortega C. Kok K. Blanco-Gutierrez I. Helfgott A. (2013). *A Handbook for the Participatory Process in ROBIN: Development of Methods for Local Stakeholder Meetings.* Wageningen, Netherlands: University Press of Wageningen.

[B116] Vaske J. J. Kobrin K. C. (2001). Place attachment and environmentally responsible behavior. *J. Environ. Educ.* 32 16–21. 10.1080/00958960109598658

[B117] Watson D. Clark L. A. Tellegen A. (1988). Development and validation of brief measures of positive and negative affect: the PANAS scales. *J. Pers. Soc. Psychol.* 54 1063–1070. 10.1037/0022-3514.54.6.1063 3397865

[B118] Whitmarsh L. (2011). Scepticism and uncertainty about climate change: dimensions, determinants and change over time. *Glob. Environ. Chang.* 21 690–700. 10.1016/j.gloenvcha.2011.01.016

[B119] Williams D. R. Vaske J. J. (2003). The measurement of place attachment: validity and generalizability of a psychometric approach. *For. Sci.* 49 830–840.

[B120] Witte K. (1992). Putting the fear back into fear appeals: the extended parallel process model. *Commun. Monographs* 59 329–349. 10.1080/10410236.2012.708633 23330860

[B121] Wittmann M. Sircova A. (2018). Dispositional orientation to the present and future and its role in pro-environmental behavior and sustainability. *Heliyon* 4:e00882. 10.1016/j.heliyon.2018.e00882 30386830 PMC6205297

[B122] Xexakis G. Trutnevyte E. (2021). Empirical testing of the visualizations of climate change mitigation scenarios with citizens: a comparison among Germany, Poland, and France. *Glob. Environ. Chang.* 70:102324. 10.1016/j.gloenvcha.2021.102324

[B123] Zlatev M. Pahl S. White M. (2010). Perceived risk and benefit for self and others as predictors of smokers’ attitudes towards smoking restrictions. *Psychol. Health* 25 167–182. 10.1080/08870440802372449 20391213

