# Supplementary material for: Co-created Future Scenarios as a Tool to Communicate Sustainable Development in Coastal Communities in Palawan, Philippines
Source: Front Psychol. 2021 Nov 22;12:627972. doi: 10.3389/fpsyg.2021.627972 (PMC8645572; doi:10.3389/fpsyg.2021.627972)
Supplement: Supplementary file 1 [file Data_Sheet_1.docx]

**Supplementary Document 1. Business as Usual Scenario**

**Instructions (via Power Point and Lead Researcher):**

“Create a stereotypical family from Taytay (husband, wife, kids)

What is their name? Age? Hair colour? Hobbies? Profession? Family? Likes? Dislikes? Clothes?

Where do they live? What are their struggles in everyday life?”

*Lead researcher developed a detailed picture of the local family together with the participants on the flip chart.*

“Now think about this family from Taytay in 15 years’ time. How will their life look like?”

*Prompt cards were spread on the tables to facilitate the discussion. Prompt cards mentioned various ecosystems (mangroves, corals, etc) as well as economic sectors (fisheries, tourism, etc) that evolved during previous stakeholder meetings.*

# **Title: Neneng and Dodoy: Living the Taytayano dream?**

*NB: Scenarios have been created in the local language (Tagalog) and were then back translated to English by the local researchers*

The world is so much more beautiful in the morning in this area of the town. Dodoy appreciated every single day to wake up at the coast of Taytay bay with the sound of the waves crashing to the shore. Living with Neneng, their two daughters, Jackie and Jessa and three sons, Jackson, Jacob and Dodoy Jr have been very fulfilling. He shuffled through the hearth to build a fire, searched for the kettle and filled it with water to boil. As he filled his mug, he felt Neneng’s arms wrapping around his chest saying: “Good morning, Palangga. (Good morning, Love.)”. Dodoy turned around and offered her a cup of warm coffee while his thoughts wandered to the increasing number of fishermen fishing in the same fishing ground with him.

Neneng prepared the breakfast, fish and rice, for their children, they have to be nourished and ready for school. Dodoy sorts his nets, hooks and lines. He plans to fish in the bay this morning before going to their farm to gather some eggs and vegetables to sell and for dinner. It was a usual day, Dodoy started the engine of his boat and sailed to the bay, hopeful for today’s catch. However, after a few minutes while the boat travels away from the shore, boats of other fishers are appearing on the horizon, all competing for the dwindling fish grounds in Taytay Bay. Neneng stayed at home to clean up, wash clothes, open her small Sari-Sari store and waited for her friends for a late morning chitchat.

Dodoy decided to steer through the mangrove area instead of fishing in the open sea. He passed through several cut and dead mangroves and thought about getting some “tamilok” later this morning. In his mind, concerns were returning on how things would turn out if the mangroves would be further destroyed. What would happen to his family then? Will he still catch enough fish for them? Will he still pass through these trees when he gets older? Will he be able to compete with other fishermen with more advanced gears and larger vessels? He hopes so. But what if he won’t? He needs another source of income, maybe a better boat? Maybe he can join Timo’s business on live fish trade. Or maybe, in God’s will, he will have a pearl farm and Neneng’s store will be bigger? Dodoy and Neneng are responsible for their future and the future of their children. Hence, discovering alternative ways of livelihood and riding the tide of progress has to be their chosen path.

Back at home, Neneng, Sabel and Cecil are having some juicy gossip about Imelda’s son who got jailed because of illegal drugs. Aside from the economic growth that tourism and technology are bringing to Taytay, crime, drug addiction and pollution are steadily creeping into in the municipality. Neneng is concerned: What if in desperate need for money to raise their family, Dodoy might also go into illegal activities? If he get arrested, what will happen to their future? Catching up with her friend’s topic she was surprised that they switched focus and were now talking about politics and the upcoming elections! Sabel exclaimed, “Palawan is going to be divided into three provinces and Taytay is going to be the capital town in the future- Or a city perhaps! I also heard that they want to federalize our country! It means we can maximize our resources more and decide for ourselves!”. Neneng then said, “Yes, it’s great but if we won’t let our voices be heard, politicians might forget about us. Fishermen are the poorest here in the Philippines and if our sector will be neglected, even worst scenario will happen!” Neneng further thought about what the future beholds. “What if one of the children went into politics?” Later that day Sabel had more news: new Pearl Farms will be established in Taytay throughout the next 10 years and today they are publishing training opportunities and job openings. Curious, Neneng closed her store and went to the market place. Maybe there are opportunities to source funds for the education of her children.

Tired with the day’s fishing, Dodoy went back ashore and sold his whole catch to Edgar, a middle man selling the fish for a much higher price to the tourist region El Nido. Instead of spending a few more hours to earn more money as a tricycle driver, he went to Taytay’s cockpit arena. Last time he lost a lot of money here which lead to conflicts with his wife. Today, however, was a more lucky day. On the way back home, he passed by his rice farm and quickly checked on the golden grains. While following his routine on the field, he ruminated about the secret he is keeping from his family for a long time now. Aside from engaging in illegal fishing, he is also regularly joining some gambling and cockfighting. He stops his thoughts by convincing himself that in the first place, he is doing these things for his family.

Later that night, when the children were safely tucked, Neneng and Dodong went out to sit in the wooden bench near the shore. Dodong wrapped his arms around Neneng and dreamt of the future, that if, maybe, if things would just continue to be just like this, business-as-usual, they would be known as a model family in their barangay, balancing a couple of different livelihoods. However, the ecosystem on which they are relying is in severe danger. Increasing numbers of fishermen, illegal settlers, mangrove cutting, pollution and the high number of tourists causes problems. Both have mixed feelings about what the future might hold for the next generations. Partly optimistic, partly fearful they are looking into the future.

In 10 years, things will be different. Dodoy will be a successful fisherman, owning a big fishing boat. He will participate in the government programs managing the fishing quotes. Neneng’s small store will be a big one, she will be busy with managing it and has no time for chit-chat. Their neighbor’s life will also change by the increase of tourists in their city.

However, despite the economic progress in Dodoy and Neneng’s life, Taytay’s environment will suffer – mangroves will be exhausted, waters polluted and the garbage will be worse than before. Some environmentally protected areas will be converted into urban centres because of political manipulation and the poor and displaced members of their town will be even more poorer, some of them even steal food to eat.

Dodoy and Neneng will realize that education, diligence and perseverance was able to combat poverty and change the future for themselves but a lot more could have been done for the community. Governments should be more sensitive and inclusive and Taytay should be protecting their environment in the midst of the quest for progress.

**Supplementary Document 2. Worst Case Scenario**

**Instructions:**

“You are a passionate environmentalist/ economist/ policy maker/ social worker and you had great plans for Taytay.

Looking back after 15 years, everything went wrong. The worst case happened…

What happened?

Tipp: Focus on your priorities first. Later, we discuss with the other subgroups.

🡪 Use colours, drawings, narratives, text…”

**Title: The Future of Taytay Starts with You**

*NB: Scenarios have been created in the local language (Tagalog) and were then back translated to English by the local researchers*

Once upon a time in the northern part of Palawan, there was a young boy named Dodoy. Young Dodoy was born and raised in the beautiful island of Taytay. One day, Dodoy’s parents talked about their plans to send Dodoy to the business school in Manila. Dodoy’s parents were involved in the live reef fish business in Taytay and want their only son to study business too. Since Dodoy is an obedient child, he followed his parents’ wish. Dodoy was very sad to leave his lovely hometown; he already missed his friends in school and the delicious seafood. He will miss the clean and colorful environment and the crystal clear waters. Dodoy promised his parents that he will study hard, find a job, earn money and comeback.

Ten years later, Dodoy is still in Manila. While driving he listened to the radio. He heard that another beach resort will be constructed in Taytay and will be featured in an international magazine. Worried about his hometown and because he has not been hearing from his parents in a while Dodoy called his Mother and asked for the updates. His mother told him “Yes son, you’ve heard it right, the newly-elected mayor had signed the contract of approval recently. Your father is still in prison because of the cyanide he was purchasing for our business. I have no job anymore. Instead, a capitalist from China took over all small businesses in the bay. This is already the month of December but it is too hot, I can feel that the climate is changing. Our new mayor does not listen to the reports of illegal activities in our area. The fishing grounds are declining, effective monitoring and enforcement of the environmental laws and regulations remains a common problem here. The electric bills have gone up and because of the long blackouts we experience right now, this is why you did not hear anything from us. Dodoy promised to continue working hard and to send some money every month.

A few months later, travelled back to Taytay for the first time since he left. Along the way, he notices many malnourished, street children and some pregnant women carrying a child on the left while holding one on the other. Dodoy’s mother has died because of a new epidemic disease in their area while his father also died in prison. Arriving at his hometown it strikes him that the once beautifully painted, clean and functioning health centers were now dusty and worn-out. He does not find any old friend because most of them were working abroad as domestic helpers. Most of the market establishments were closed and prices of his favorite bread have gone up incredibly high. The once barren graveyards were crowded as diseases and lack of medications was not available in their area. Illegal mining spread over the region, uncontrolled agriculture caused the last bits of forest to be cut down. Taytay bay was full of container ships, tourist boats and large fishing vessels, many of them without a legal flag. Along the coast, multiple resorts for cheap tourism were built, many of them already collapsing again. On many places of the city, waste dumps could be found, pollution not only the watersheds but also covering the city in a penetrating smell. No mangroves were lefty along the coastline of Taytay region and the marine environment was heavily damaged by illegal fishing activities, sedimentation and pollution: all seagrass beds and corals were found dead. The water on the beaches became so murky that swimming was impossible. Tourists and businesses carelessly continued littering.

During his visit, Dodoy could not take any bath since the fresh water supply stopped, sources of water were not functioning because of lack of maintenance. He called the local government about the issue, but they only blamed the previous government instead of trying to find solutions. While sitting on the porch of the veranda of his family house Dodoy broke out in tears and said to himself “How did this beautiful Taytay, become so destroyed? I don’t want my kids to grow up in this setting.”

**Supplementary Document 3. Best Case Scenario**

**Instructions:**

“You have the power to change Taytay’s future to the better and fulfil the dreams you have for this place. But first, which superhero are you?”

Participants are encouraged to think of their favourite superhero and tell their group members.

Nest, followed a two-step process:

“If you could change things in Taytay for good, how would it look like?

- Stick your ideal future on notes to the map
- What would need to happen to get there?

🡪 New leaders? New regulations? More awareness? More/less tourism…?”

**Title: The Taytay Avengers**

*NB: Scenarios have been created in the local language (Tagalog) and were then back translated to English by the local researchers*

Have you ever wondered, if the environment is threatened, who will come to save it? This is how our story will unfold today. It starts from the peaceful town of Taytay, The Star of the North.

Taytay is a place full of bountiful blessings from God and nature. Blue seas rich in fish and marine animals, Green mountains with lots of trees that provide fresh water to the rivers and streams. The people are contented of their daily lives and everything is simple. Until one day, the mayor of the town announced that they will embrace the new technology arising. They will create buildings made of quarried materials, they will automate their transportation and more people will be staying in the town center. Communication will be using mobile phones and television will be available in households. Education and health care will be improved by making more buildings and hiring more staffs.

The Taytayanons were delighted by the idea. They’ve come to the town to experience the new way of life. As many people flock to Taytay, the demand for commodities grew and the population is rising. New technology enables farmers to increase seeding and harvesting. Fishers begin to double their yield from the modern technology of fish finders and bigger nets. Everyone was enjoying at first.

But then the population grew even bigger. Immigrants came to see the opportunities for fishing and farming. Mangrove areas were cut down to serve as housing areas. More people have access to communication and technology and knew the demand for products from the wild, so they began hunting. Tourists flocked and everyone shared the goodness and resources of nature.

A decade had passed. However, natures’ gifts for Taytay were not able to hold on and greed struck men. They began to seize the opportunity of paying greedy mercenaries to continue logging of the rain and mangrove forests. The increased people’s taxes and stole them. They allowed illegal activities to prosper.

As taxes soared along with prices of commodities, Taytayanons became desperate. They tried to exhaust the environment for more income.. The poor who earn less became sick without money for medicine. The capitalists became richer. And many resorted to criminal acts such as thievery.

On the continuing growth of evil, a group of students came to a meeting and discussed for themselves if this is the future they want to have. One day, their prayers were answered as a white dove came from the sky and grant them powers. Powers of change and compassion for the people and the environment. One by one, they transformed and called themselves, the Taytay Avengers.

The leader of the Taytay Avengers was Master of Jaguar, a stealthy and intelligent half human/half jaguar. He brings the power to build the capacity of Taytay citizens for competitive sustainable tourism, which will attract global investment, while also supporting local employment, demonstrating its global/local competitiveness.

The superhero with most intellect was the Goddess of Venus, providing the power of environmental education, that can improve productivity and sustainability. She specializes in sustainable agriculture, fisheries, and tourism, with her aim of making the citizens of Taytay highly educated.

The team is joined by The Magicians, an elusive mysterious group of aboriginal magic-practitioners from the forest, their powers are to preserve knowledge about ancient medicine, to rehabilitate natural environments, including coastal clean-ups and to plant mangroves. The Magicians work closely with the Goddess of Venus to combine conservation and sustainable resource-use activities.

Robin Hood uses his magic bow to hunt down and shoot dishonest government officials and poachers, his magic arrow turns them into honest, unwavering and educated role models, who are incorruptible and support cooperation between the citizens and government officials.

The Superman Triplets are powerful and strong, with the strength to carry out sustainable development and the building of new infrastructure to support this growth. They also convey strong morals so act as role models to make disciplined, spiritual citizens of Taytay, while also using their strength to help law enforcers hunt and prevent illegal activities.

The team is now ready. They set the plan on how to cure the people. Each one contributed and vowed to do their best, for the people and the environment.

The Team first intercepted illegal activities in the forests. The Magicians first displayed their powers by showing people each medicinal plant and gave them instructions on how to use them and to properly. The Goddess of Venus showed herself in the community. She used her charming powers to gather people and made them listen to the importance of the environment. They explained the relationship between illegal fishing and deforestation to climate change. They gave demonstrations and carefully taught the people how not to exhaust nature when doing farming and fishing. The also made better ways to do tourism while preserving the environment. They encouraged people to clean their surroundings. They taught proper disposal of wastes, even human waste and to recycle non-biodegradable materials. After all the training, they all went to the shores and revisited the mangrove areas, they were then taught on proper mangrove planting and coral reef protection.

While The Goddess of Venus and The magicians worked hand-in-hand, Robin Hood and Master Jaguar prepared for a battle. They know that facing government officials is a tough encounter. As Robin Hood came to investigate, he prepared his bow and arrow and shoot the evil within those government officials. His skills were extraordinary: he freed them of their spells. When the mayor came into good senses, they were given the best education by the Goddess of Venus and eventually, they realized the effects of what they had done. Robin Hood then ushered them to the people, and the mayor ordered for the seizure of all poachers and illegal loggers. All of the wild animals imprisoned were released. Master Jaguar advised the Mayor to offer help and cash incentives to them to have a change of heart and leave all the illegal activities. The criminals form the past now turned into town cleaners, Bantay Dagat, Bantay Kalikasan, construction workers and tour guides.

To ensure all this, Master Jaguar used his might to capacitate Taytayanons for competitive sustainable tourism, which will attract global investment. They helped them give proper training in responsible tourism and responsible development to ensure the building of new structures while giving less stress to the environment. Schools and hospitals were built without any trace of corruption.

With the combined powers of the Taytay Avengers, Taytay became home to good, honest citizens, who help to preserve the natural environment, while also supporting sustainable development focused on tourism, fisheries, and agriculture.

**Supplementary Document 4. Stakeholder Scenario Workshop**

The Blue Communities project encompasses three regions across Palawan (Puerto Princesa, Aborlan and Taytay) of which one (Taytay) was selected as a focus area for this study. In collaboration with 23 local stakeholders from various sectors such as the local government, NGOs, representatives of fisheries, aquaculture and tourism, three scenarios were developed (for details see Table 1 below). During the workshop, participants collaborated in small groups. There was a total of four groups in which people have been collaborating throughout the day. The first scenario, Business as Usual (BAU), represents the continuation of the current situation and its developments into the future (see Supplementary Document 1). Under this scenario, the current local problems of the local community such as illegal fishing, mangrove cutting and commercial fishing vessel intrusion were narrated and the most likely future outcomes in the next fifteen years were depicted. The concept of Business as Usual has been introduced and discussed in plenum. Subsequently, the participants were asked to describe a typical family from Taytay (husband, wife, kids) including details such as their name, age, hair color, hobbies, profession and more. These details have been depicted in form of a sketch on a flip chart. As a next step, the small groups elaborated on this families’ challenges in everyday life as well as how these might develop in 15 years’ time. After 30 minutes of in-group discussion, participants listed their ideas in a flip chart per group. Representative for each group reported their output (Figure 4.1 in this document). Under this scenario, the current local problems of the community, such as illegal fishing, mangrove cutting and commercial fishing vessel intrusion were discussed.

**
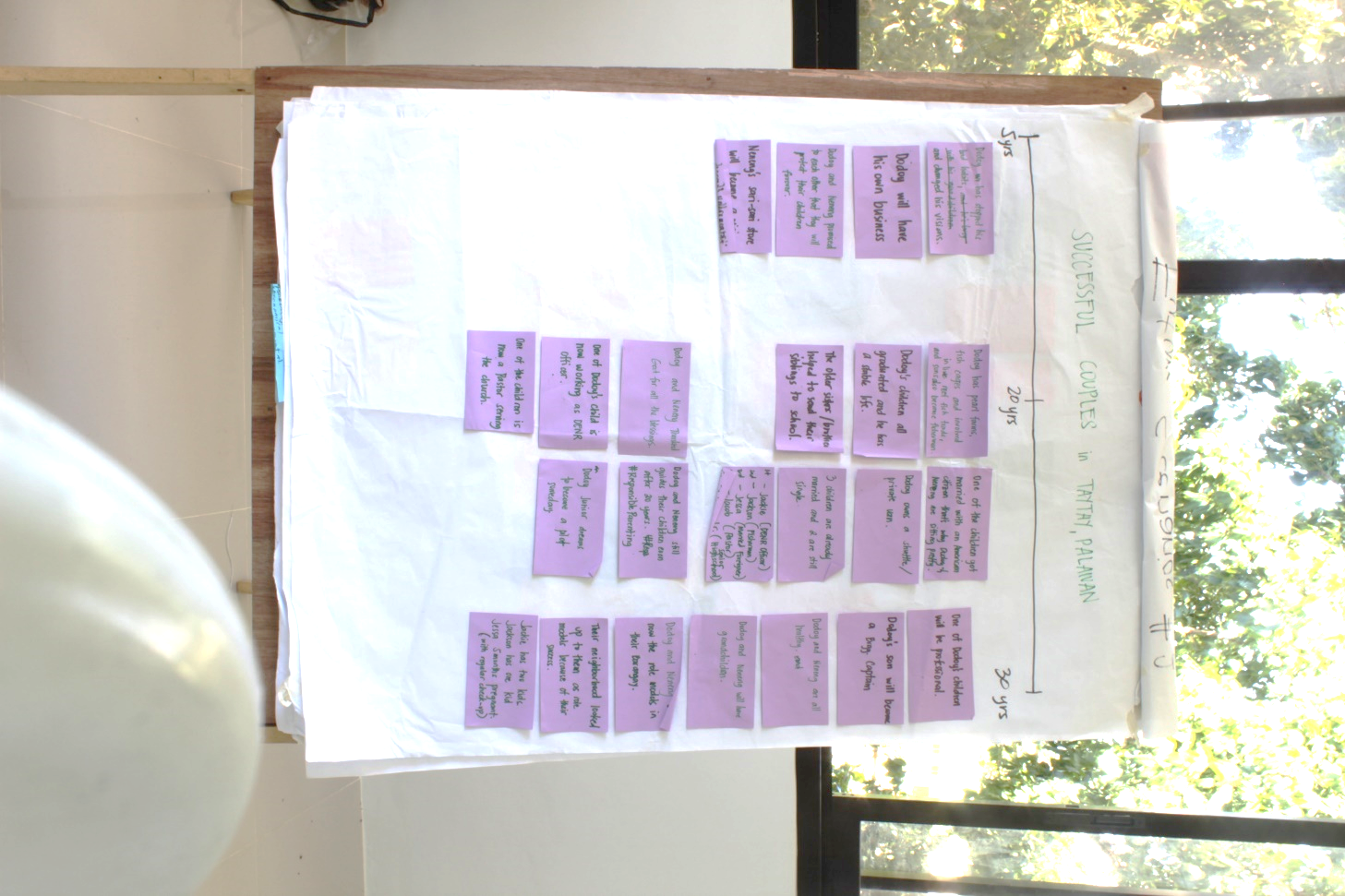
**

**Figure 4.1**: Scenario development workshop output; Business as Usual Scenario

The second part of the workshop was the development of the Worst Case Scenario (WC, see Supplementary Document 2). It was aimed to represent the least desirable future. For this scenario, each group was asked to focus on the worst possible development of only one sector, the environment, society, the economy or politics. Subsequently, the groups merged these negative prospects within each sector to one comprehensive worst case scenario (Figure 4.2 in this document). In this scenario, current developments have been driven towards a negative extreme through the narrative: steeply declining fish stocks and dead coral reefs, malnourished children, epidemic diseases and ubiquitous pollution.

**
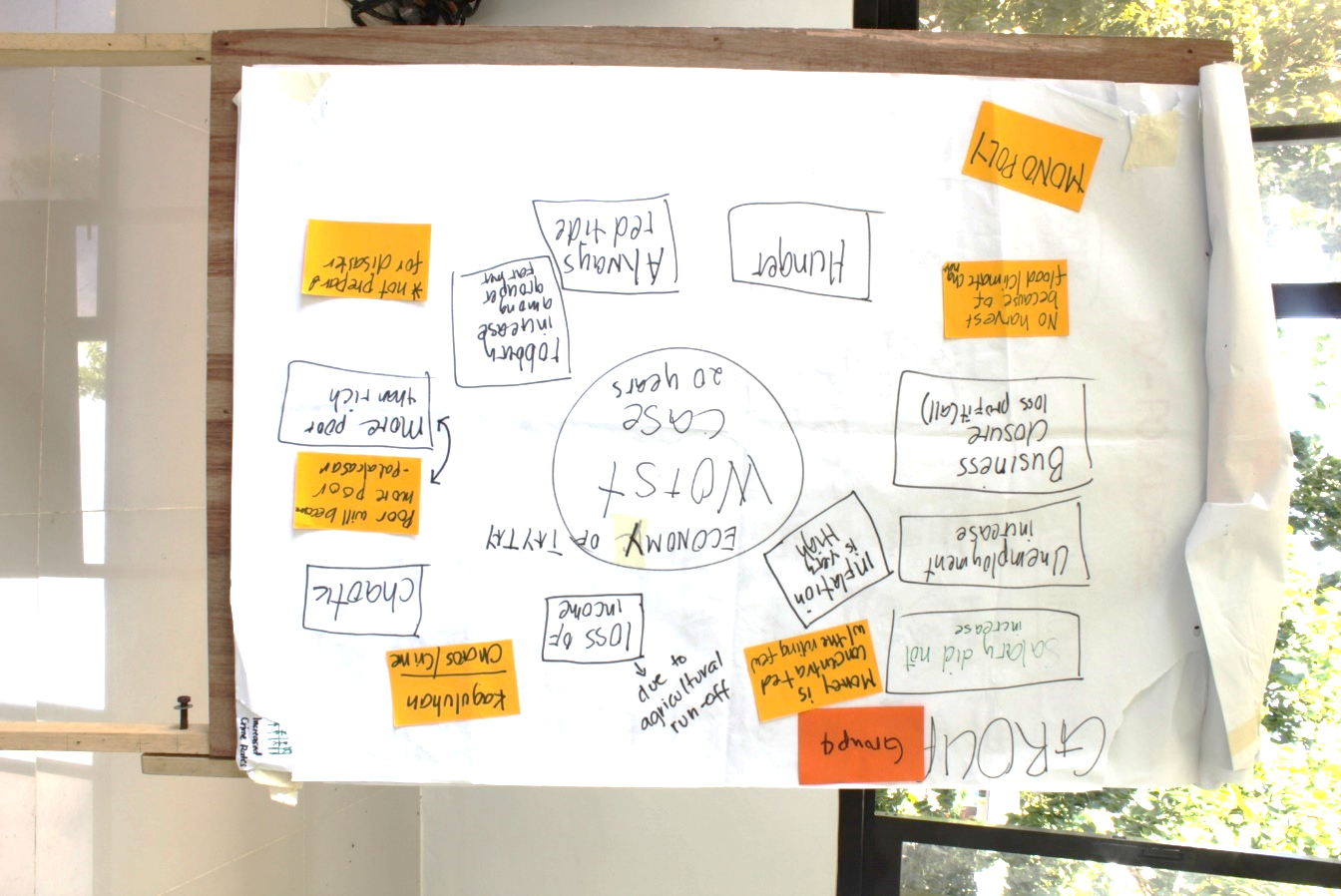
**

**Figure 4.2**: Scenario development workshop output; Worst Case Scenario

The Best Case Scenario (BC) was developed to depict the most desirable future possible outcomes and, in addition, to identify potential pathways to realize these. It was developed by encouraging the participants to think as superheroes with the capacity to change the future of Taytay. The rationale behind the superhero strategy is that it allows participants to think “out of the box” and beyond what they think is realistic. The participants were asked to map their ideal future in consideration to what they could do in the role of a superhero to change things for the better. The key question was: If you could change Taytays future for the better, how would it look like and what would you do (Figure 4.3 in this document)? Easy life in a simple environment was narrated in this scenario. The best case scenario showed that the most desirable outcomes would be achieved with improved management interventions, such as good performing officials, mangrove restoration, coral reef protection, and sustainable fishing practices.


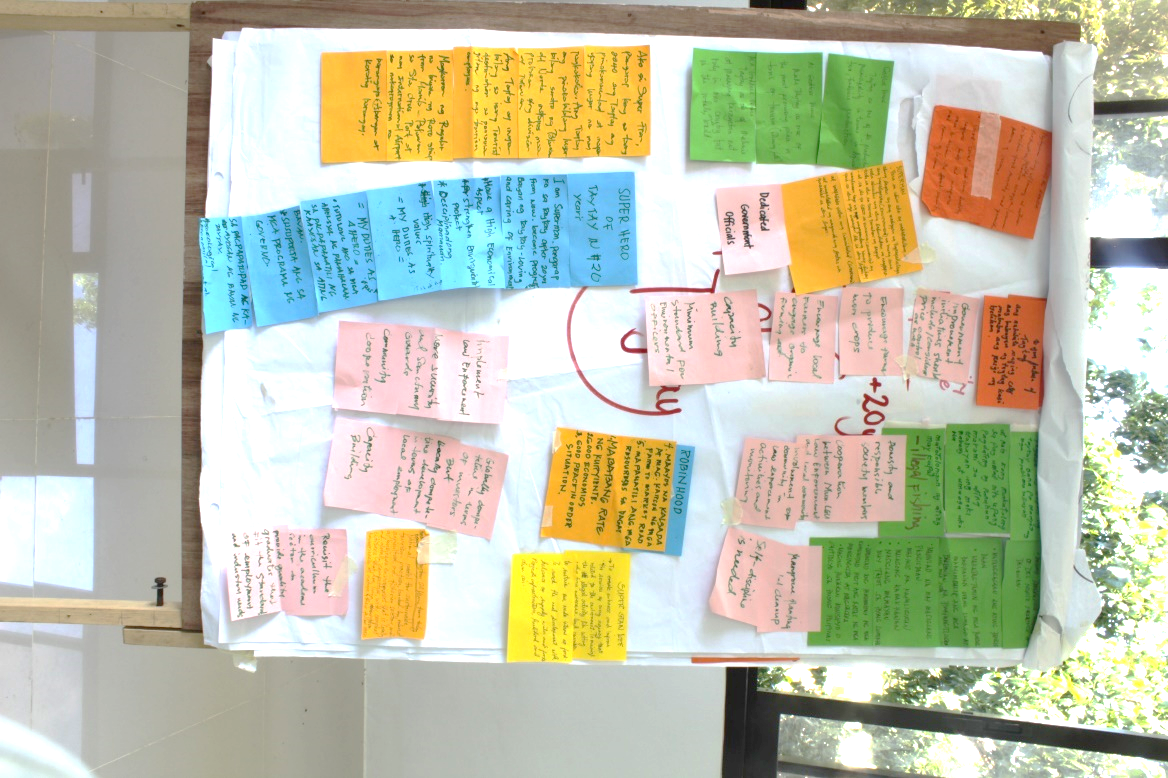


**Figure 4.3**: Scenario development workshop output; Worst Case Scenario

All three scenarios depicted a version of the future in 15 years’ time, following the recommendations to use a human time horizon (Pahl, 2010; Pahl et al., 2014). The development of the scenarios followed the principle of participatory research by Green et al. (2003), according to which the research process is gradually co-shaped by researchers and participants, and scenarios were elicited with group work manuals adapted from Mansfield (2018) (exact task instructions in the Appendix).

The rich volume of information provided by the stakeholders was collaboratively synthesized by the research team and turned into three coherent narratives (see Supplementary Files 1-3). As a common element, all three narratives revolved around one protagonist, representing a stereotypical local family.

**Table 1**. Workshop participants, *N*=23, and represented authority

| Representative | Male | Female |
| --- | --- | --- |
| Local Legislation Member | 1 |  |
| Local Environmental Officer | 1 |  |
| Local Tourism Officer | 1 |  |
| Non-Government Organization |  | 1 |
| Fishing Community Representative | 2 |  |
| Local Agriculture Officer |  | 1 |
| Local Government Head |  |  |
| International Academe | 1 | 1 |
| Local Academe | 5 | 3 |
| Local Community Representative | 4 | 2 |
| Total | 15 | 8 |

**Supplementary Document 5. PART 1**

**ID________**

**Dear valued participant,**

Thank you for helping with this research. We highly value your time and input.

Please let us know if you have any questions at any point.

There are no right or wrong answers; we are interested in your views.

First, we would like you to answer some general questions about yourself.

| 1. **What is your gender?**  \|  \| Male \| \| --- \| --- \| \|  \| Female \| \|  \| Don't want to disclose \| |  | 1. **Where are you from?**  \|  \| Taytay \| \| --- \| --- \| \|  \| Palawan \| \|  \| Somewhere else in the Philippines \| \|  \| Somewhere outside the Philippines \| |
| --- | --- | --- | --- | --- | --- | --- | --- | --- | --- | --- | --- | --- | --- | --- | --- | --- |
| 1. **What is your age, in years?**   ___________________________________ |  | 1. **What grade are you?**   ___________________________________ |

1. **How connected do you feel to…**

Please circle a number to show how connected you feel to the following places.

|  | Not connected at all |  |  |  | Very connected |
| --- | --- | --- | --- | --- | --- |
| … Your city/village | 1 | 2 | 3 | 4 | 5 |
| … Your region | 1 | 2 | 3 | 4 | 5 |
| … Your country | 1 | 2 | 3 | 4 | 5 |
| … The world as a whole | 1 | 2 | 3 | 4 | 5 |

**ID_______**

1. **To what degree do you agree with the following statements?**

|  | **Strongly disagree** | **Disagree** | **Neutral** | **Agree** | **Strongly agree** |
| --- | --- | --- | --- | --- | --- |
| I don’t feel responsible for the problems of my community | 1 | 2 | 3 | 4 | 5 |
| I think it is difficult to do something for my community as an individual | 1 | 2 | 3 | 4 | 5 |
| In the near future, I want to engage more in work that helps my community | 1 | 2 | 3 | 4 | 5 |
| I consider how things might be in the future, and try to influence those things with my day to day behaviour. | 1 | 2 | 3 | 4 | 5 |
| Often I engage in a particular behaviour in order to achieve outcomes that may not result for many years. | 1 | 2 | 3 | 4 | 5 |
| I am willing to sacrifice my immediate happiness or wellbeing in order to achieve future outcomes. | 1 | 2 | 3 | 4 | 5 |
| When I make a decision, I think about how it might affect me in the future. | 1 | 2 | 3 | 4 | 5 |
| My behaviour is generally influenced by future consequences. | 1 | 2 | 3 | 4 | 5 |

1. **How do you feel when you think about the future?**

| **Thinking about the future makes me feel…** | **Strongly disagree** | **Disagree** | **Neutral** | **Agree** | **Strongly agree** |
| --- | --- | --- | --- | --- | --- |
| Worried | 1 | 2 | 3 | 4 | 5 |
| Empowered to do something | 1 | 2 | 3 | 4 | 5 |
| Hopeful | 1 | 2 | 3 | 4 | 5 |
| Scared | 1 | 2 | 3 | 4 | 5 |
| Angry | 1 | 2 | 3 | 4 | 5 |
| Curious | 1 | 2 | 3 | 4 | 5 |

**ID______**

1. **How many years do you think ahead when you think of the future of your community?**

| 1 day | 1 month | 1 year | 5 years | 10 years | 15 years | 25 years | 50 years |
| --- | --- | --- | --- | --- | --- | --- | --- |
| O | O | O | O | O | O | O | O |

1. **How important are the following goals for your community?**

|  | Not very important |  | Neutral |  | Very important |
| --- | --- | --- | --- | --- | --- |
| A stable economy, Money | 1 | 2 | 3 | 4 | 5 |
| A happy society, Wellbeing | 1 | 2 | 3 | 4 | 5 |
| A healthy environment, Nature | 1 | 2 | 3 | 4 | 5 |

1. **What are your personal hopes for your future and the future of your community?**

____________________________________________________________________________________

____________________________________________________________________________________

____________________________________________________________________________________

1. **What are your personal worries for your future and the future of your community**?

____________________________________________________________________________________

____________________________________________________________________________________

____________________________________________________________________________________

**Thank you for your participation.**

**Questions?** Isabel.richter@plymouth.ac.uk

**Supplementary Document 5. PART 2**

**ID_________________**

1. **How connected do you feel to…**

Please circle a number to show how connected you feel to the following places.

|  | Not connected at all |  |  |  | Very connected |
| --- | --- | --- | --- | --- | --- |
| … Your city/village | 1 | 2 | 3 | 4 | 5 |
| … Your region | 1 | 2 | 3 | 4 | 5 |
| … Your country | 1 | 2 | 3 | 4 | 5 |
| … The world as a whole | 1 | 2 | 3 | 4 | 5 |

1. **To what degree do you agree with the following statements?**

|  | **Strongly disagree** | **Disagree** | **Neutral** | **Agree** | **Strongly agree** |
| --- | --- | --- | --- | --- | --- |
| I don’t feel responsible for the problems of my community | 1 | 2 | 3 | 4 | 5 |
| I think it is difficult to do something for my community as an individual | 1 | 2 | 3 | 4 | 5 |
| In the near future, I want to engage more in work that helps my community | 1 | 2 | 3 | 4 | 5 |
| I consider how things might be in the future, and try to influence those things with my day to day behaviour. | 1 | 2 | 3 | 4 | 5 |
| Often I engage in a particular behaviour in order to achieve outcomes that may not result for many years. | 1 | 2 | 3 | 4 | 5 |
| I am willing to sacrifice my immediate happiness or wellbeing in order to achieve future outcomes. | 1 | 2 | 3 | 4 | 5 |
| When I make a decision, I think about how it might affect me in the future. | 1 | 2 | 3 | 4 | 5 |
| My behaviour is generally influenced by future consequences. | 1 | 2 | 3 | 4 | 5 |

**ID_______**

1. **How do you feel when you think about the future?**

| **Thinking about the future makes me feel…** | **Strongly disagree** | **Disagree** | **Neutral** | **Agree** | **Strongly agree** |
| --- | --- | --- | --- | --- | --- |
| Worried | 1 | 2 | 3 | 4 | 5 |
| Empowered to do something | 1 | 2 | 3 | 4 | 5 |
| Hopeful | 1 | 2 | 3 | 4 | 5 |
| Scared | 1 | 2 | 3 | 4 | 5 |
| Angry | 1 | 2 | 3 | 4 | 5 |
| Curious | 1 | 2 | 3 | 4 | 5 |

1. **How many years do you think ahead when you think of the future of your community?**

| 1 day | 1 month | 1 year | 5 years | 10 years | 15 years | 25 years | 50 years |
| --- | --- | --- | --- | --- | --- | --- | --- |
| O | O | O | O | O | O | O | O |

1. **How important are the following goals for your community?**

|  | Not very important |  | Neutral |  | Very important |
| --- | --- | --- | --- | --- | --- |
| A stable economy, Money | 1 | 2 | 3 | 4 | 5 |
| A happy society, Wellbeing | 1 | 2 | 3 | 4 | 5 |
| A healthy environment, Nature | 1 | 2 | 3 | 4 | 5 |

**ID_______**

| 1. **Which activity did you engage in today?** | \|  \| 1 \| Painting Business as Usual Scenario \| \| --- \| --- \| --- \| \|  \| 2 \| Painting Worst Case Scenario \| \|  \| 3 \| Painting Best Case Scenario \| |
| --- | --- | --- | --- | --- | --- | --- | --- | --- | --- | --- |

1. **How much did you enjoy the activity you were engaging in?**

|  | Not at all |  | Neutral |  | Very much |
| --- | --- | --- | --- | --- | --- |
| I enjoyed the activity I was involved in. | 1 | 2 | 3 | 4 | 5 |

1. **What are your personal hopes for your future and the future of your community?**

____________________________________________________________________________________

____________________________________________________________________________________

____________________________________________________________________________________

1. **What are your personal worries for your future and the future of your community**?

____________________________________________________________________________________

____________________________________________________________________________________

____________________________________________________________________________________

**Thank you for your participation.**

**Questions?** Isabel.richter@plymouth.ac.uk
